# Multiple-Strain Infections of Human Cytomegalovirus With High Genomic Diversity Are Common in Breast Milk From Human Immunodeficiency Virus–Infected Women in Zambia

**DOI:** 10.1093/infdis/jiz209

**Published:** 2019-05-03

**Authors:** Nicolás M Suárez, Kunda G Musonda, Eric Escriva, Margaret Njenga, Anthony Agbueze, Salvatore Camiolo, Andrew J Davison, Ursula A Gompels

**Affiliations:** 1 Medical Research Council–University of Glasgow Centre for Virus Research, United Kingdom; 2 Pathogen Molecular Biology Department, London School of Hygiene and Tropical Medicine, United Kingdom; 3 Virology Laboratory, University Teaching Hospital, Lusaka, Zambia; 4 Birkbeck College, University of London, United Kingdom

**Keywords:** human cytomegalovirus, high-throughput sequencing, breast milk, target enrichment, viral genomics, bioinformatics

## Abstract

**Background:**

In developed countries, human cytomegalovirus (HCMV) is a major pathogen in congenitally infected and immunocompromised individuals, where multiple-strain infection appears linked to disease severity. The situation is less documented in developing countries. In Zambia, breast milk is a key route for transmitting HCMV and carries higher viral loads in human immunodeficiency virus (HIV)–infected women. We investigated HCMV strain diversity.

**Methods:**

High-throughput sequence datasets were generated from 28 HCMV-positive breast milk samples donated by 22 mothers (15 HIV-infected and 7 HIV-negative) at 4–16 weeks postpartum, then analyzed by genome assembly and novel motif-based genotyping in 12 hypervariable HCMV genes.

**Results:**

Among the 20 samples from 14 donors (13 HIV-infected and one HIV-negative) who yielded data meeting quality thresholds, 89 of the possible 109 genotypes were detected, and multiple-strain infections involving up to 5 strains per person were apparent in 9 HIV-infected women. Strain diversity was extensive among individuals but conserved compartmentally and longitudinally within them. Genotypic linkage was maintained within hypervariable UL73/UL74 and RL12/RL13/UL1 loci for virus entry and immunomodulation, but not between genes more distant from each other.

**Conclusions:**

Breast milk from HIV-infected women contains multiple HCMV strains of high genotypic complexity and thus constitutes a major source for transmitting viral diversity.

## Teaser

In an investigation of human cytomegalovirus in breast milk from mothers in Zambia, samples from HIV-infected women were found to contain multiple viral strains of high genotypic complexity. Milk thus presents a major source for transmitting viral diversity to infants.

**(See the major Article by Suarez et al, on pages 781–91.)**

Human cytomegalovirus (HCMV) is a major coinfection in human immunodeficiency virus (HIV)–infected people, in whom, as in other immunocompromised individuals such as transplant recipients, it contributes to morbidity and mortality. HCMV is also the most frequent congenital infection, causing adverse neurodevelopment, including hearing loss, microcephaly, and neonatal morbidity. Postnatal infection generally occurs via milk in breastfeeding populations and is usually asymptomatic. However, it has been linked to morbidity, especially in preterm or underweight infants, and, in recent population studies, to adverse developmental effects, especially in association with HIV exposure in developing countries [1–4]. The most severe HCMV infections in transplant recipients, whether due to primary infection, reinfection, or reactivation from latency, can result in severe or end-organ diseases such as retinitis, pneumonitis, hepatitis, and enterocolitis [5]. Few studies of HCMV diversity, transmission, and epidemiology have been conducted in relation to developing countries, including those having a high burden of endemic HIV.

HCMV has a double-stranded DNA genome of 236 kbp containing at least 170 protein-coding genes [6]. Diversity among strains is low overall, except in several hypervariable genes that exist as distinct, stable genotypes. These genes encode proteins that are particularly vulnerable to immune selection, including virus entry glycoproteins, other membrane glycoproteins, and secreted proteins. The recombinant nature of HCMV strains was first identified in serological surveys and then in genomic studies, and is a key consideration for vaccine development [7–16]. However, understanding the pathogenic effects of HCMV diversity is at an early stage [17–19], and is limited by the fact that most analyses have focused on a few hypervariable genes characterized by polymerase chain reaction (PCR)–based genotyping [7, 12, 20]. This approach is relatively insensitive to the presence of minor strains in multiple-strain infections, which may have more serious outcomes.

High-throughput sequencing studies at the whole-genome level have started to facilitate a broader view of HCMV diversity, but most have involved isolating the virus in cell culture, which is prone to strain loss or mutation, or have depended on direct sequencing of PCR amplicons generated from clinical samples [11, 14, 16, 21]. Recent studies have avoided these limitations by using target enrichment to enable direct sequencing of strains present in clinical samples, most of which originated from patients in developed countries with congenital or transplantation-associated infections [11, 22–24]. Here, we use this and new methods to examine HCMV strain diversity in a developing country by analyzing breast milk from women in Zambia, who constitute an HIV-endemic population in sub-Saharan Africa, where we have previously demonstrated the negative developmental effects of early infection of infants with HCMV, particularly alongside HIV exposure [1, 3].

## METHODS

### Patients and Samples

Anonymized breast milk samples were collected with informed consent as a substudy of the Breast Feeding and Postpartum Health study conducted at the University Teaching Hospital, Lusaka, Zambia, as approved by the ethical committees of the University Teaching Hospital and the London School of Hygiene and Tropical Medicine. This substudy included 28 HCMV-positive breast milk samples donated from one or both breasts by 15 HIV-infected and 7 HIV-negative mothers at 4 and/or 16 weeks postpartum (Table 1 and Supplementary Table 1 [rows 3–6]) [3].

**Table 1.** Characteristics of Donors, Samples, and Datasets

Donor^a^	HIV Status	Breast Sample	Weeks Postpartum	HCMV Load, ge/mL^b^	Dataset	Strains^c^ Detected
158	Negative	Left	16	818 244	158L16	2
166	Negative	Left	16	282 252	166L16	1
193	Negative	Right	16	215 217	**193R16** ^d^	**1**
232	Negative	Right	4	470 150	232R4	2
239	Negative	Right	4	4 752 875	239R4	1
263	Negative	Left	4	5 285 775	263L4	1
280	Negative	Right	4	7 505 536	280R4	1
141	Positive	Right	16	319 888	**141R16** ^e^	**2**
154	Positive	Left	16	2 195 856	154L16	2
173	Positive	Right	16	349 532	**173R16** ^d^	**5**
**174**	Positive	Left	16	371 027	**174L16** ^d^	**3**
**174**	Positive	Right	16	642 516	**174R16** ^d^	**3**
181	Positive	Left	4	1 972 365	181L4	2
**243**	Positive	Left	16	643 895	**243L16** ^e^	**2**
**243**	Positive	Right	4	65 511 020	**243R4** ^d^	**1**
**243**	Positive	Right	16	795 092	**243R16** ^d^	**1**
248	Positive	Right	4	441 679	**248R4** ^d^	**1**
258	Positive	Right	4	610 613	**258R4** ^d^	**2**
**259**	Positive	Left	16	2 366 193	**259L16** ^d^	**3**
**259**	Positive	Right	16	5 053 047	**259R16** ^d^	**3**
264	Positive	Left	16	388 519	**264L16** ^d^	**2**
277	Positive	Right	16	250 377	**277R16** ^d^	**2**
**278**	Positive	Left	16	3 751 776	**278L16** ^d^	**2**
**278**	Positive	Right	4	294 246 272	**278R4** ^d^	**2**
**278**	Positive	Right	16	4 370 800	**278R16** ^d^	**2**
281	Positive	Right	4	20 291 530	**281R4** ^d^	**2**
283	Positive	Right	16	274 391	**283R16** ^d^	**1**
288	Positive	Right	4	31 574 022	**288R4** ^d^	**3**

Abbreviations: HCMV, human cytomegalovirus; HIV, human immunodeficiency virus; ge, genomic equivalent.

^a^Donor IDs in bold and underlined are sequential or from paired tissue samples.

^b^Median loads are higher in HIV-positive compared to negative and also in week 4 compared to week 16 as shown previously [3].

^c^Number of strains detected are from Table 3, only those meeting quality thresholds noted are in bold, with the original data from the Supplementary Tables 1 and 3.

^d^Met all quality thresholds.

^e^Met all quality thresholds except that unique fragment coverage depth was 10–20 rather than ≥20 reads/nt.

### DNA Extraction and Viral Load Quantification

DNA was extracted from 200 µL breast milk using a QIAamp DNA mini kit (Qiagen), and viral DNA load measured using an HCMV gB TaqMan assay on an Applied Biosystems 7500 fast real-time PCR system (Applied Biosystems), as described (Table 1 and Supplementary Table 1 [row 7]) [3].

### High-Throughput DNA Sequencing

The SureSelect version 1.7 target enrichment system (Agilent) was used to prepare sequencing libraries (Supplementary Table 1 [rows 8–10]) [22]. These were sequenced using a MiSeq (Illumina) with version 3 chemistry generating original datasets of paired-end reads of 300 nucleotides (nt) (Table 1 and Supplementary Table 1 [rows 11–12]).

### Phylogenetic Analysis

UL73 and UL74 genotypes [7, 12] were investigated in 243 different HCMV strains with complete genome sequences available [24]. MEGA 6.06 [25] was used to generate muscle-derived amino acid sequence alignments and phylogenetic trees based on the Jones–Taylor–Thornton model and discrete gamma distribution with 5 categories.

### Strain Characterization Using Sequence Motifs

Original datasets were quality-checked and trimmed using Trim Galore (http://www.bioinformatics.babraham.ac.uk/projects/trim_galore/; length = 21, quality = 10 and stringency = 3) (Supplementary Table 1 [row 13]). Bowtie2 [26] was used to remove reads mapping to the Genome Reference Consortium Human Reference 38 sequence, quality-checked and trimmed to create purged datasets (Supplementary Table 1 [row 14]). Dataset quality parameters were set on thresholds described in the Results (Supplementary Table 1 [rows 19–23]) [24].

The number of genotypes was analyzed by counting reads containing conserved, genotype-specific sequence motifs or their reverse complements. One short motif (14 nt) for each UL73 genotype and 3 short motifs (12–13 nt) for each UL74 genotype were identified by initially examining nucleotide sequence alignments and polymorphism plots derived from the 163 HCMV complete genome sequences in GenBank Release 211 (15 December 2015). Motif conservation was confirmed in the 243 genome set as described [24] plus 383 UL73 and 72 UL74 single-gene sequences available in GenBank, which originated from various tissues, including milk (the UL73/74 single gene set only), and various locations worldwide, including Zambia (single gene set only) [3, 7, 12]. The sequences of the short motifs are listed in Table 2 with their frequency of occurrence.

**Table 2.** Short Motif Sequences in UL73 and UL74

Gene	Position^a^	Genotype	Motif Sequence^b^	Sequences, No.^c^	Occurrences, No.^c^	Frequency, %^c^
UL73	5′	G1	GCGTATCAACTACC	121	121	100
		G2	GTGTGTCGACGAGT	53	53	100
		G3A	GCGTGTCAACAAGC	104	104	100
		G3B	GTGTATCAACGGTA	47	47	100
		G4A	GCACCTTAACAACC	114	113	99
		G4B	ACACCTCAACGACC	55	55	100
		G4C	GCACCTCAACAACC	39	38	97
		G4D	ACGCCTCAACAACC	93	92	99
UL74	5′	G1A	AAACGACWATTT	47	43	91
		G1B	AAAAGGATATCT	60	60	100
		G1C	AAAGGGAACCTT	19	19	100
		G2A	AACCTATTCCTT	27	27	100
		G2B	AGAGCGACATAT	38	38	100
		G3	CGAGCCAGGATT	66	64	97
		G4	AAACAGGTGATT	19	19	100
		G5	TGTCTACATCAT	38	38	100
UL74	C	G1A	CCTTGTGGTACTG	47	47	100
		G1B	TCTTGCGGTACGG	60	60	100
		G1C	TCTTGTGGTACAG	19	19	100
		G2A	TCGTGTGGCGCAG	27	27	100
		G2B	CCTTGCGGTACAG	38	38	100
		G3	TCTTGTGGCACTG	66	66	100
		G4	TCCTGTGGYACGA	19	19	100
		G5	CCTTGYGGCACAG	38	38	100
UL74	3′	G1A	TATTACTACCGCC	47	47	100
		G1B	TGTTACTACCACC	60	60	100
		G1C	GGTTACCACCAGC	19	19	100
		G2A	TGTTACCACCACC	27	27	100
		G2B	TGTTACAACCACC	38	38	100
		G3	TGCTACCACCACT	66	66	100
		G4	TCCTATTGTCCCA	19	19	100
		G5	TGCTACCGCTGCT	38	38	100

^a^5′, toward the 5′ end of the protein-coding region; C, in the central part of the protein-coding region; 3′, towards the 3′ end of the protein-coding region; in reference strain Merlin, the UL73 5′ motif is located at 104–117 nucleotides (nt) in UL73 (408 nt; G4D), and the UL74 5′, C and 3′ motifs are located at 206–217, 443–454, and 906–918 nt, respectively, in UL74 (1419 nt; G5).

^b^UL73 and UL74 are transcribed rightward and leftward, respectively, in the human cytomegalovirus genome; the sequences are presented 5′-3′ in relation to the direction of transcription; international union of pure and applied chemistry nucleotide codes are used.

^c^The total number of sequences in the 243 genome set plus single-gene sequences, followed by the number and percentage of these sequences possessing the motif; one UL74 intergenic recombinant (BE/23/2010) was excluded. This provides a measure of motif sensitivity.

In addition to counting short motifs (Supplementary Table 1 [rows 25–56]), long motifs (20–24 nt) at one per genotype were counted in UL73 and UL74 and a further 10 hypervariable genes (RL5A, RL6, RL12, RL13, UL1, UL9, UL11, UL120, UL146, and UL139) (Supplementary Table 1 [rows 58–166]) [24]. Long motifs identifying common gene-disrupting mutations in 3 genes (RL5A, UL111A, and US9) [24] were also counted (Supplementary Table 1 [rows 167–174]). Strain numbers were estimated from long motif counts using thresholds described in the Results (Supplementary Table 1 [row 17]).

### Variant Analysis

Replacement of one strain by another as the major population (genotype switch) in compartmental or longitudinal samples from the same individual, were investigated by variant analysis [21, 27]. The original datasets were quality checked using FASTQC (http://www.bioinformatics.babraham.ac.uk/projects/fastqc/), trimmed to not less than 100 nt using Trimmomatic [28], optimized using VelvetOptimiser parameters (http://www.vicbioinformatics.com/software.velvetoptimiser.shtml), and assembled de novo using Velvet [29], producing contigs that were ordered by reference genome mapping using ABACAS [30]. The resulting contigs were verified by reference mapping using BWA [31] and SAMtools/BCFtools [32]. GATK [33] was used for indexing, mapping and variant calling, defining variant nucleotides as follows: prevalence <50%, overall read depth ≥50, average nucleotide quality ≥30, variant frequency ≥1% for read depths >1000 and >10% for read depths 50–1000, and minimum SNP depth ≥10. Artemis [34] was used for visualization.

### Data Deposition

The human purged datasets were deposited in the European Nucleotide Archive under project number PRJEB31143 (Supplementary Table 1 [row 15]). Complete genome sequences were assembled as described [22] and deposited in GenBank (Supplementary Table 1 [row 16]) under accessions MK290742–MK290744 and MK422176.

## RESULTS

### Dataset Assessment

In a recent study, we highlighted the importance of monitoring dataset quality produced directly from clinical material by target enrichment and high-throughput sequencing [24]. We implemented this here by assembling datasets against the reference strain Merlin genome (GenBank accession AY446894), noting numbers of matching HCMV reads (Supplementary Table 1 [line 19]), and deriving 2 parameters: (1) percentages of matched HCMV reads in the dataset and (2) percentages of the reference genome represented (Supplementary Table 1 [lines 20–21]). Also, since sequencing methodology is highly PCR based, the number of HCMV fragments producing the data was monitored by additional parameters: (3) coverage depth of the reference genome by all HCMV reads, and (4) coverage depth of the reference genome by reads generated from unique HCMV DNA fragments (Supplementary Table 1 [lines 22–23]).

Quality threshold values were set at (1) ≥50%, (2) ≥95%, (3) ≥1000 of the total fragment reads/nt, and (4) ≥20 unique fragment reads/nt. Eighteen datasets generated from 13 women met all 4 criteria, and 2 datasets (141R16 and 243L16) met criteria (1) to (3) but exhibited lower values (10–20) for criterion (4). These 20 datasets (from one HIV-negative woman and 19 from 13 HIV-infected women) (Table 1 and Supplementary Table 1 [row 11]) are analyzed further.

### Genotypic Structure of UL73/UL74

**Figure 1.**
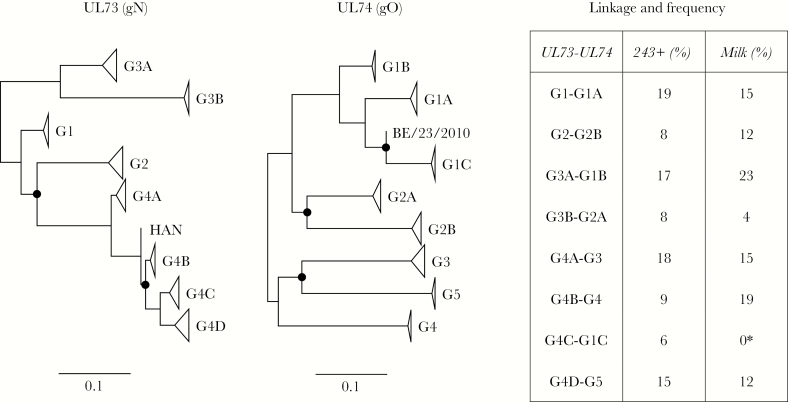
Unrooted phylogenetic trees for UL73 and UL74 based on amino acid sequences derived from 243 genome sequences, and a summary of genotypic linkages and frequencies. The site coverage cutoff value was 95%, leaving 134 sites in the UL73 tree (log likelihood, –1117.87) and 435 sites in the UL74 tree (log likelihood, –4153.86). Branch point robustness was inferred from 100 bootstrap replicates, and values of <70% are denoted by filled circles. Genotype branches are collapsed, and the numbers of substitutions per site, are shown by the scale. The UL73 sequence of strain HAN and the UL74 sequence of strain BE/23/2010 did not fall into the genotypes. The linkages between UL73 and UL74 genotypes are listed, followed by the frequencies of UL73 genotypes in the 243 genome sequences plus 383 single-gene sequences (243plus; 626 in total; Table 2), and the frequencies of the deduced linkages in the samples (milk; 26 in total; Table 3). The frequency of each genotype in the milk set was not significantly different (above *P* = .05) from that in the 243 plus 383 single gene set, as determined by random subsampling analysis (10 000 samplings of 26 genotypes from the set of 626). *Although no examples of this linkage were present in the datasets at levels in excess of the thresholds, at least one patient (258) was infected at subthreshold levels by a relevant strain (Supplementary Table 1).

Our previous study involving Sanger sequencing of single HCMV genes in breast milk samples obtained at multiple time points postpartum pointed to the presence of multiple strains [3]. We extended this here by using sequence differences between the genotypes of hypervariable genes across the genome to characterize the strains represented in the datasets. We focused first on UL73 and UL74, as our earlier work had shown that these adjacent genes are markedly hypervariable, are almost always genotypically linked, grouping into 8 genotypes, also identified in milk samples [3, 7, 12]. The nucleotide sequences were extracted from the set of 243 genome sequences for which complete genome sequences were available [24] and analyzed phylogenetically (Figure 1). This confirmed the existence of 8 genotypes for each gene (Table 2), their strong linkage (only 7 recombinants were noted), and high levels of intergenotypic diversity and low levels of intragenotypic diversity as observed initially in small datasets [7, 12]. In the UL73 phylogeny, a single G4B strain (HAN; GenBank accession number KJ426589) fell outside the genotypes due to 3 nucleotide differences that are characteristic of G4A strains and probably represent homoplasies. In the UL74 phylogeny, a single strain (BE/23/2010; GenBank accession KP745697) fell outside the genotypes potentially from intragenic recombination between G1C and G1A. The distances between genotypes and the branching patterns in the 2 phylogenies also supported our previous inference that an ancestral recombination event had given rise to the linkage between UL73 G4C and UL74 G1C [12].

**Table 3.** Genotypes and Haplotypes Assigned to Datasets

Donor	Dataset	Strains^a^	Genotypes^b^											
			RL5A	RL6	RL12	RL13	UL1	UL9	UL11	UL73	UL74	UL120	UL146	UL139
193	193R16^c,d^	1	1	3	8	8	8	4	1	3A	1B	2B	12	3
141	141R16	2	1, 6	2, 3	2, 4B	2	2, 4	2, 3	5, 6	2, 3A	1B, 2B	4B	2, 12	3, 8
173	173R16	5	1, 2, 6	2, 3, 4	1B, 3, 4B, 6, 8	1, 6, 8	1, 6, 8	2, 4, 6, 7, 9	1, 3, 5	1, 3A, 4A, 4B	1A, 1B, 3, 4	1A, 4B	3, 7, 9, 12	1A, 1B, 3, 7
174	174L16	3	2	2, 4	1B, 6	1, 6, 10	1, 4, 6	4, 6	1, 4	1, 2, 4A	2B, 3, 4	2B, 3A	7, 9	5, 7
174	174R16	3	1, 2	1, 2, 3, 4	1B, 6	1, 6, 10	1, 6	4, 6, 7	1, 4	2, 4A	1B, 2B, 3	2B, 3A	7, 9	5, 7
243	243L16^c^	2	1	6	2, (10)	(1), 2	2	3	6	4B	4	2A	8, 9	7
243	243R4^c,d^	1	1	6	2	2	2	3	(1), 6	4B	4	2A	8	7
243	243R16^c^	1	1	6	2	2	2	3	6	4B	4	2A	8	7
248	248R4^c,d^	1	1	1	4A	4A	4	1	1	3B	2A	4B	[5]	4, (7)
258	258R4^c^	2	6	3	3	3	3	3	6	3A, (4A)	1B, (3)	(2A), 2B	(2), 9	5
259	259L16	3	1, 2	2, 3, 4	1A, 6, 8	1, 6, 8	1, 6, 8	1, 3, 6	1, 4, 6	1, 2, 4B	1A, 2B, 4	4B	1, 9, 12	2, 8
259	259R16	3	1, 2	2, 3, 4	1A, 6, 8	1, 6, 8	1, 6, 8	1, 3, 6	1, 4, 6	1, 2, 4B	1A, 2B, 4	4B	1, 9, 12	2, 8
264	264L16	2	1	1, 2	8, 10	8, 10	10	8	4, 7	3A, 4B	4	1A, 3A	10	3
277	277R16	2	1	3	4A, 6	4A, 6	4, 6	6, 9	1, 4	1, 4A	1A, 3	3A, 4A	1, 9	3, 4
278	278L16^c^	2	(2), 6	3, (4)	(1A), 9	(1), [9]	(1), 9	(1), 9	(1), 6	3A, (4D)	1B, (5)	(3A), 4B	8, (9)	2, (2)
278	278R4^c^	2	(2), 6	3, (4)	(1A), 9	(1), [9]	(1), 9	(1), 9	(1), 6	3A, (4D)	1B, (5)	(3A), 4B	8, (9)	2, (2)
278	278R16^c^	2	(2), 6	3, (4)	(1A), 9	(1), [9]	(1), 9	(1), 9	(1), 6	3A, (4D)	1B, (5)	(3A), 4B	8, (9)	2, (2)
281	281R4^c^	2	(1), 6	3	1A, (6)	1	1, (10)	4, (7)	1	4D	(1B), 5	4B	11	5, (7)
283	283R16^c,d^	1	[2]	4	4B	[4B]	4	2	5	3A, (3B)	1B	3A	1	5
288	288R4	3	1, 2	3, 4	6, 7	6, 8	5, 6, 8	4, 6, 9	1	1, 4B, 4D	1A, 4, 5	2B, 4B	1, 3	4, 5

^a^Determined using long motifs for 12 genes (Supplementary Table 1).

^b^Genotype (G) prefix omitted; round brackets indicate an assigned minority genotype; multiple genotypes with none in round parentheses indicate that majority and minority genotypes could not be distinguished; square brackets indicate a single mismatch in the motif.

^c^Datasets from which haplotypes were assigned.

^d^Datasets from which complete genome sequences were derived.

### Genotyping Using Sequence Motifs

Having established a comprehensive view of UL73 and UL74 hypervariation, we developed short motifs capable of identifying individual genotypes. These consisted of a single motif near the 5′ end of each UL73 genotype and 3 separate motifs near the 5′ and 3′ ends and in the central region of each UL74 genotype, and successfully genotyped the majority of sequences used in the phylogenetic analyses (Table 2). We then extended the analysis to a further 10 hypervariable genes, using a single, long, nonredundant motif for each genotype to improve discrimination.

The original datasets were trimmed (to create trimmed datasets) or purged of human reads and trimmed (to create purged datasets). The relative frequencies of individual genotypes were then estimated by counting motifs in each dataset with threshold requirements (Supplementary Table 1 [lines 25–56 and 58–166], respectively). Purging human reads had little effect, except when short motifs were used with datasets containing a significant proportion of residual nonviral reads. The UL74 5′ motif offered the least accurate genotypic discernment in such samples, perhaps from its minimal length (12 nt). The number of strains in each sample was scored from the purged datasets using the long motifs with threshold requirements (Table 3 and Supplementary Table 1 [row 17]). A genotype was considered to be present when represented by >25 reads and >5% of the total number of reads detected for all genotypes of that gene, and the number of strains was scored as being the greatest number of genotypes detected using long motifs for at least 2 genes. Thus, strains present at <5% were unlikely to score. There was a high degree of congruence between the results obtained using short and long motifs with datasets meeting the quality thresholds (Supplementary Table 2).

### Strain Complexity in HIV-Infected Women

The majority of HIV-infected women (11/13) were infected by multiple HCMV strains (Table 3 and Supplementary Table 1). The mode was at least 2 strains, and one woman was infected by 5 strains. In the dataset meeting quality thresholds, the only HIV-negative woman was infected by a single strain. This was also indicated in the datasets from the 6 other HIV-negative women, but these were below quality thresholds, partly from lower viral loads, and not compared further. Even among this small cohort, 89 of the 109 possible genotypes for the 12 hypervariable genes were detected. It was possible to assign with confidence fully linked genotypes (haplotypes) to 8 strains represented in 11 datasets from 7 donors, on the basis of complete genome sequences (4 datasets) or the presence of a single strain or major and minor strains (when the former was highly predominant) in multiple-strain infections (Table 3). Consideration of all the other datasets from multiple-strain infections where both major and further minor strains could be identified, allowed haplotypes to be assigned to a further 12 strains, but with less confidence, 20 total (Supplementary Table 3).

Genotypic linkage was detected only in 2 loci where recombination has been shown to occur rarely, namely, those containing the 2 respective sets of adjacent, hypervariable genes UL73/UL74 [12, 17, 35] and RL12/RL13/UL1 [11, 16]. The overall frequencies of UL73/UL74 genotypes in the milk samples were not significantly different from those in the 243 genome set plus the 383 single-gene sequences (Tables 1 and 3). Comparisons to only the 243 genome set, which does not include milk or African samples, showed some evidence for increased proportions of UL73/UL74 G4B-G4 and RL12/RL13/UL1 G2-G2-G2 in milk (*P* = .001 and *P* = .02, respectively), but case-controlled cohorts are required to confirm.

**Figure 2.**
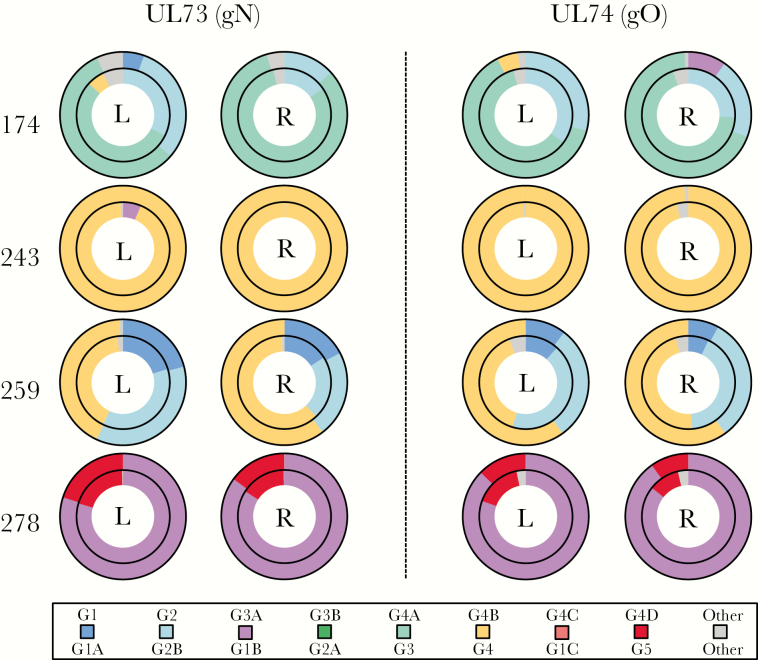
UL73 and UL74 genotypes in milk samples collected from the left (L) and right (R) breasts of 4 human immunodeficiency virus–infected donors at 16 weeks postpartum (Table 1). The inner and outer rings show the results obtained using short and long motifs, respectively. Short motif 3′ was used for UL74 (Table 2). The color key for genotypes is shown at the foot. Reads that did not meet the inclusion criteria for genotyping are shown as “other.”

The use of 3 short motifs in UL74 facilitated an examination of intragenic recombination, and confirmed that strain BE/23/2010 is a recombinant with a G1C motif near the 5′ end and G1A motifs in the central region and near the 3′ end. In addition, compartmental stability was revealed by the use of both short and long motifs, in the form of genotypic conservation in samples from both breasts of 4 HIV-infected women (Figure 2). Small differences may be accounted for by minor strains present at levels nearing the detection threshold. Longitudinal stability was observed in 2 donors (243 and 278) with samples taken at weeks 4 and 16 postpartum (Table 3); small differences in one (243) were probably due to threshold effects. This stability also showed in variant analysis, which demonstrated the absence of genotype switches in all donors.

Finally, additional long sequence motifs were used to investigate whether any strains contained gene-disrupting mutations detected previously in the 243 genome set, and resulting in pseudogenes [24]. Such mutations are more common in certain genes, most frequently in UL9, RL5A, UL1, RL6, US9, and UL111A [14, 16, 24]. The use of motifs representing 3 mutations in RL5A (present in 37 members of the 243 genome set), 2 in US9 (35 members) and one in UL111A (5 members), demonstrated the presence of the RL5A and US9 mutations, but not that in UL111A, encoding viral interleukin 10 (Supplementary Table 1 [rows 167–174]).

## DISCUSSION

Analysis of HCMV genomes directly from clinical samples is necessary for characterizing infectious natural populations while avoiding the mutational artefacts arising from laboratory adaptation to cell culture. Target enrichment has proven successful in this regard [11, 22, 24], but accurate genome analysis can be confounded by multiple strains, particularly in immunosuppressed groups in whom additional complexity may accumulate by reinfection or reactivation [17, 21, 24]. We have shown previously that HIV-infected women in sub-Saharan Africa have higher HCMV loads in breast milk than HIV-negative women, and that this is associated with adverse infant development [1, 3]. However, genomic studies of HCMV in milk samples or, indeed, samples from Africa, are scarce. We examined milk because of its importance in HCMV transmission, with the aim of understanding strain diversity and the burden of infection in HIV-infected (immunosuppressed) mothers, which may affect their infants. The sequence datasets were generated from 28 samples donated by 22 women, and 20 datasets from 14 women meeting quality thresholds were analyzed.

The analysis focused on counting reads containing motifs specific to the genotypes of hypervariable genes. Short motifs were developed initially for sensitive characterization of UL73 and UL74, which encode glycoproteins N and O (gN and gO), respectively, and then long motifs were used for further resolution of these 2 genes and 10 others. Since, as shown further here, UL73 and UL74 are linked and behave as a single genotype, haplotypes could not be determined using solely the short motifs (Supplementary Table 2) [7, 12]. However, mapping 3 short motifs to each UL74 genotype was uniquely useful for detecting intragenic recombination. The use of long motifs in a larger number of genes allowed increased resolution and also haplotype determination. These were less compromised by residual human reads in the datasets, but more susceptible to mismatches in target genomes (Table 3).

Genotypic and haplotypic complexity in this small cohort was remarkable. Most (82%) of the genotypes possible in the 12 hypervariable genes were detected, and 85% of the HIV-infected donors were infected by multiple strains. The level of multiple strain infection exceeded that in previous cohort analyses, including congenitally infected and transplantation patients from developed countries [22, 24]. Each of the 20 fully characterized haplotypes identified was unique in this cohort and in the set of 243 strains, in which most strains (223) are also unique [24]. These observations testify to the huge number of HCMV haplotypes that may exist, possibly exceeding that related to immune diversity, as was recognized long before the high-throughput sequencing era [7–9, 12, 13, 15]. No evidence emerged for the existence of novel African genotypes, consistent with the view that HCMV genotypes are distributed throughout the world, although their relative prevalence may vary [7, 8, 12].

Strain composition in individual women was essentially stable, both compartmentally (in milk samples from both breasts) and longitudinally (at 4 and 16 weeks postpartum). This indicates that the strains detected were present in the donor prior to viral reactivation, which peaks at 4 weeks in breast tissue during lactation [3]. Leukocyte infiltrates have been characterized during this period [36], and may be the source of reactivated virus. It may also be that the strains in blood differed from those in milk, but this was not investigated. A saliva-based study conducted in Uganda by PCR and antibody assays indicated that HCMV secretion was induced in seropositive mothers after exposure to their HCMV-excreting children [37]. However, even though multiple-strain infections were common in the cohort and opportunities for fresh infection existed at home or in the hospital because all the mothers were HCMV-infected and had young children at home, there was limited evidence for reinfection or reactivation with new strains during the 4- to 16-week period postpartum. These observations differ from those made in developed countries in transplantation patients. A proportion of transplant-associated infections involve multiple strains, and these exhibit substantial longitudinal dynamism [22, 24] and are also associated with increased viral loads and the pathological outcomes of HCMV disease [18, 20]. The contrasting observation, that most congenital or postnatal infections involve single strains [24, 38], suggests that only certain strains cross the placenta or are transmitted by breast milk, urine, or saliva, perhaps due to the competence of a few virions to establish infection [38, 39]. This also implies that the HIV-infected women were exposed to a high burden of HCMV superinfection.

Whole-genome analyses and earlier PCR-based studies showed a high degree of linkage within the UL73/UL74 [7, 12, 24, 35] and RL12/RL13/UL1 loci [11, 24]. This is consistent with the involvement of homologous recombination during HCMV evolution, and may also reflect the functional constraints imposed on proteins that interact with each other or have interdependent functions. UL73/gN and UL74/gO are part of the viral entry complex and have roles in viral exocytosis, cellular tropism, and modulation of antibody neutralization, and the RL12, RL13, and UL1 proteins are known or suspected to be involved in aspects of immune evasion probably mediated by an immunoglobulin-like binding domain shared by these proteins and other members of the RL11 family [9, 13, 40–43]. In addition, RL13 may influence the effect of UL74 on the growth of HCMV [44]. It is possible that different genotypes of hypervariable genes, and different combinations of genotypes, provide variable growth properties leading to higher viral loads and specific pathologies. For example, UL74 genotypes differentially affect viral growth properties in vitro [45], and genotypes of UL146, which is the most hypervariable gene in HCMV and encodes a vCXCL1 chemokine, affect neutrophil chemotaxis efficiency [46]. Similarly, human genetic variation is higher in Africa than other regions and may affect susceptibility as a result of HCMV genotype-specific interactions, for example with immunoglobulin variants [47–50].

Although information on genotypes and mutants could be extracted from the datasets regardless of strain complexity, complete genome assembly was possible for only 4 datasets because of a high frequency of confounding multiple infections. To our knowledge, these are the first complete HCMV genome sequences to be determined from people living in Africa. Moreover, one of these originated from an HIV-negative woman (193) and thus represents the first from an immunocompetent adult lacking HCMV-associated pathology. Future research is likely to focus on understanding the differences in HCMV transmission in immunosuppressed and immunocompetent settings to define the interplay between viral strain and host immunotype diversity in controlling disease.

## Supplementary Data

Supplementary materials are available at *The Journal of Infectious Diseases* online. Consisting of data provided by the authors to benefit the reader, the posted materials are not copyedited and are the sole responsibility of the authors, so questions or comments should be addressed to the corresponding author.

jiz209_suppl_Supplementary_Table_1

jiz209_suppl_Supplementary_Table_2

jiz209_suppl_Supplementary_Table_3

## Notes

***Acknowledgments.*** We thank the participants and clinical staff, as managed by Dr L. Kasonka at the University Teaching Hospital, Lusaka, for facilitating the Breastfeeding and Postpartum Health Study, as directed by Professor S. Filteau, London School of Hygiene and Tropical Medicine, and for enabling the follow-up analyses. We also thank Dr J. Hughes, Medical Research Council–University of Glasgow Centre for Virus Research, for advice on bioinformatic analysis, and Drs T. Clark and J. Phelan, London School of Hygiene and Tropical Medicine, for facilitating genomics UNIX cluster access and Perl support.

***Financial support.*** This work was supported by the Commonwealth Scholarship Commission; a Bloomsbury Studentship Award; the Medical Research Council (grant number MC_UU_12014/3); and the Wellcome Trust (grant number 204870/Z/16/Z).

***Potential conflicts of interest.*** All authors: No reported conflicts of interest. All authors have submitted the ICMJE Form for Disclosure of Potential Conflicts of Interest. Conflicts that the editors consider relevant to the content of the manuscript have been disclosed.

Published as a bioRxiv preprint on 23 December 2018 and revised on 19 February 2019 (https://doi.org/10.1101/493742).
