# Supplementary material for: Multiple-Strain Infections of Human Cytomegalovirus With High Genomic Diversity Are Common in Breast Milk From Human Immunodeficiency Virus–Infected Women in Zambia
Source: J Infect Dis. 2019 May 3;220(5):792–801. doi: 10.1093/infdis/jiz209 (PMC6667993; doi:10.1093/infdis/jiz209)
Supplement: jiz209_suppl_Supplementary_Table_1 [file jiz209_suppl_supplementary_table_1.pdf]

Supplementary Table 1. Dataset characteristics and genotyping.

SUMMARY DATA[a]																											
Donor		158		166		193		232		239		263		280		141		154		173		174				181	
HIV status		Negative		Negative		Negative		Negative		Negative		Negative		Negative		Positive		Positive		Positive		Positive				Positive	
Breast sample		Left		Left		Right		Right		Right		Left		Right		Right		Left		Right		Left		Right		Left	
Time postpartum (weeks)		16		16		16		4		4		4		4		16		16		16		16		16		4	
HCMV load (genome equivalents/ml)		818,244		282,252		215,217		470,150		4,752,875		5,285,775		7,505,536		319,888		2,195,856		349,532		371,027		642,516		1,972,365	
Genome equivalent copies for library (no.)		4,091		1,411		2,152		2,351		9,506		10,572		15,011		1,599		4,392		3,495		1,855		3,213		3,945	
Pre-enrichment PCR cycles (no.)		18		18		12		18		18		12		18		18		18		12		18		18		18	
Post-enrichment PCR cycles (no.)		20		20		16		20		21		16		23		20		21		19		16		16		23	
Dataset name		158L16		166L16		193R16		232R4		239R4		263L4		280R4		141R16		154L16		173R16		174L16		174R16		181L4	
Original reads (no.)		1,771,316		1,384,030		3,405,448		2,035,362		1,736,548		2,943,760		2,358,930		2,293,074		2,262,556		2,906,542		1,833,888		2,167,500		2,763,302	
Trimmed reads (no.)		1,771,198		1,383,900		3,405,432		2,035,300		1,736,364		2,943,670		2,358,392		2,293,040		2,262,124		2,906,448		1,833,862		2,167,482		2,763,040	
Purged reads (no.)		247,328		92,562		3,303,418		688,564		319,904		132,588		1,550,084		1,991,574		787,076		2,827,534		1,759,734		1,926,042		2,545,146	
ENA purged short read accession no.		ERR3163177		ERR3163178		ERR3163179		ERR3163180		ERR3163181		ERR3163182		ERR3163198		ERR3163199		ERR3163224		ERR3163231		ERR3163232		ERR3163233		ERR3163234	
GenBank complete genome accession no.						MK290742																					
Strains (no.)		2		1		1		2		1		1		1		2		2		5		3		3		2	
ASSEMBLY DATA (STRAIN MERLIN REFERENCE)[b]		Trimmed	Purged	Trimmed	Purged	Trimmed	Purged	Trimmed	Purged	Trimmed	Purged	Trimmed	Purged	Trimmed	Purged	Trimmed	Purged	Trimmed	Purged	Trimmed	Purged	Trimmed	Purged	Trimmed	Purged	Trimmed	Purged
HCMV reads (no.)		191,423	191,417	53,193	53,193	3,017,071	3,014,727	601,083	601,082	206,712	206,712	64,835	64,831	1,333,597	1,332,519	1,771,082	1,771,081	643,552	643,552	2,567,305	2,566,526	1,586,786	1,586,777	1,742,233	1,742,190	2,242,511	2,242,508
HCMV reads (%) (1)		11	77	4	77	89	77	30	77	12	77	2	77	57	77	77	77	28	77	88	77	87	77	80	77	81	77
Coverage extent (% genome) (2)		97	97	70	70	98	98	85	85	67	67	97	97	71	71	97	97	85	85	99	99	97	97	97	97	95	95
Coverage depth (total fragment reads/nt) (3)		202	202	55	55	3,679	3,676	635	635	245	245	81	81	1,587	1,585	1,867	1,867	750	750	3,162	3,161	1,680	1,680	1,833	1,832	2,709	2,709
Coverage depth (unique fragment reads/nt) (4)		16	16	2	2	175	175	3	3	2	2	9	9	2	2	13	13	4	4	70	70	51	51	77	77	9	9
GENOTYPING DATA (SHORT MOTIF-MATCHING COUNTS)[c]		Trimmed	Purged	Trimmed	Purged	Trimmed	Purged	Trimmed	Purged	Trimmed	Purged	Trimmed	Purged	Trimmed	Purged	Trimmed	Purged	Trimmed	Purged	Trimmed	Purged	Trimmed	Purged	Trimmed	Purged	Trimmed	Purged
UL73-G1-5'	GCGTATCAACTACC	0	0	2	2	0	0	0	0	0	0	0	0	0	0	0	0	0	0	318	317	63	63	26	26	0	0
UL73-G2-5'	GTGTGTCGACGAGT	0	0	2	0	0	0	0	0	2	2	1	0	0	0	345	345	0	0	77	77	582	583	216	216	3	1
UL73-G3A-5'	GCGTGTCAACAAGC	0	0	0	0	5258	5224	0	0	0	0	96	96	0	1	1008	1009	1	0	1138	1138	60	60	28	26	1	1
UL73-G3B-5'	GTGTATCAACGGTA	0	0	0	0	0	0	1	0	0	0	0	0	0	0	0	0	0	0	0	0	0	0	0	0	0	0
UL73-G4A-5'	GCACCTTAACAACC	271	272	2	0	0	0	0	0	2	0	4	0	0	0	0	0	20	0	1730	1734	987	989	1127	1128	593	593
UL73-G4B-5'	ACACCTCAACGACC	2	1	1	1	47	47	0	0	2	1	1	0	3	3	0	0	4	0	212	212	109	109	3	3	4	4
UL73-G4C-5'	GCACCTCAACAACC	1	0	0	0	0	1	0	0	4	3	5	0	9	1	2	1	0	0	4	5	4	4	1	0	178	176
UL73-G4D-5'	ACGCCTCAACAACC	1	1	0	0	15	16	1376	1376	4	3	1	1	6	5	13	13	5	2	11	6	1	1	0	0	3	6
UL74-G1A-5'	AAACGACWATTT	21	0	172	156	65	64	16	4	23	4	33	2	1755	1746	7	5	34	1	648	648	17	14	21	21	38	40
UL74-G1B-5'	AAAAGGATATCT	73	0	33	0	5547	5457	42	15	44	3	157	56	11	8	515	502	62	2	2049	2049	3	1	51	49	25	5
UL74-G1C-5'	AAAGGGAACCTT	38	1	41	0	3	1	17	0	51	1	110	1	13	2	5	2	40	0	3	3	6	1	10	1	25	5
UL74-G2A-5'	AACCTATTCCTT	40	0	63	0	4	3	38	1	62	0	154	1	15	1	15	1	55	4	51	46	5	4	7	1	5	5
UL74-G2B-5'	AGAGCGACATAT	2	0	7	0	3	0	4	4	13	5	10	1	1	1	606	605	9	4	57	57	719	718	524	522	8	7
UL74-G3-5'	CGAGCCAGGATT	203	179	18	1	1	0	55	0	33	1	50	0	49	2	5	1	18	1	785	785	1754	1759	1021	1018	519	519
UL74-G4-5'	CGACAGGTGATT	0	1	7	0	11	7	6	1	3	0	9	1	8	0	5	7	8	3	7	4	8	3	4	1	10	7
UL74-G5-5'	TGTCTACATCAT	32	1	34	1	20	18	37	2	22	2	84	1	30	5	8	2	29	4	12	11	6	2	13	3	20	19
UL74-G1A-C	CCTTGTGGTACTG	5	5	8	3	20	2	2	1	1	0	15	0	4	3	6	3	1	0	903	902	12	11	102	102	5	5
UL74-G1B-C	TCTTGCGGTACGG	1	0	0	0	4650	4628	3	3	0	0	116	113	1	0	1979	1979	2	2	2216	2216	34	34	109	109	7	6
UL74-G1C-C	TCTTGTGGTACAG	0	0	9	0	0	1	9	2	3	1	15	0	0	1	2	0	10	0	1	2	125	123	4	3	9	9
UL74-G2A-C	TCGTGTGGCGCAG	9	0	0	0	3	1	2	1	4	0	3	0	15	13	3	4	17	15	50	47	1	4	4	3	8	9
UL74-G2B-C	CCTTGCGGTACAG	4	0	5	0	0	1	11	5	0	0	12	0	3	1	1674	1675	6	0	107	110	909	907	630	630	0	0
UL74-G3-C	TCTTGTGGCACTG	200	171	14	2	16	14	16	0	18	2	36	0	3	0	12	6	46	1	894	896	1168	1165	853	850	11	11
UL74-G4-C	TCCTGTGGYACGA	3	0	6	1	11	7	5	1	12	0	25	1	7	1	6	5	11	2	36	35	8	7	25	19	3	3
UL74-G5-C	CCTTGYGGCACAG	25	0	12	0	13	9	252	238	23	0	64	1	20	14	9	3	46	2	10	8	15	9	12	3	12	3
UL74-G1A-3'	TATTACTACCGCC	1	0	70	68	2	1	4	0	1	0	3	1	8	7	0	0	2	0	578	577	4	3	23	23	1144	1144
UL74-G1B-3'	TGTTACTACCACC	7	0	22	1	5164	5147	10	0	11	1	79	54	12	6	288	286	56	4	2104	2104	2	2	4	2	9	10
UL74-G1C-3'	GGTTACCACCAGC	3	0	3	0	37	35	9	1	6	1	12	1	5	3	4	3	10	3	10	10	10	7	5	4	39	39
UL74-G2A-3'	TGTTACCACCACC	50	5	30	3	64	55	49	5	354	314	86	2	86	5	15	11	22	3	123	122	14	9	22	6	137	128
UL74-G2B-3'	TGTTACAACCACC	10	2	2	1	15	15	15	1	2	1	30	0	1	2	461	458	18	1	247	246	558	557	387	384	20	19
UL74-G3-3'	TGCTACCACCACT	233	156	39	0	8	4	25	2	49	1	44	0	7	1	6	3	974	954	1494	1492	965	963	1022	1022	697	699
UL74-G4-3'	TCCTATTGTCCCA	17	0	5	0	2	3	8	0	680	657	27	0	2	1	11	0	4	0	545	542	47	47	30	30	2	2
UL74-G5-3'	TGCTACCGCTGCT	105	0	110	2	31	19	1006	860	58	0	168	2	6	4	12	4	299	220	23	15	2	1	19	14	18	15
GENOTYPING DATA (LONG MOTIF-MATCHING COUNTS)[c]		Trimmed	Purged	Trimmed	Purged	Trimmed	Purged	Trimmed	Purged	Trimmed	Purged	Trimmed	Purged	Trimmed	Purged	Trimmed	Purged	Trimmed	Purged	Trimmed	Purged	Trimmed	Purged	Trimmed	Purged	Trimmed	Purged
RL5A-G1	ATTTTATCATTAAACCCCAATATT	2	2	0	0	5268	5268	1119	1132	0	0	0	0	4194	4194	595	595	4024	4020	1679	1680	4	3	75	75	3709	3709
RL5A-G2	ATGTCCACGTTGAATTCAACTTAG	0	0	0	0	0	0	0	0	0	0	0	0	0	0	0	0	0	0	787	787	538	537	1024	1024	0	0
RL5A-G3	GCTTCCAAGTTGACGTAAGATCGT	0	0	0	0	0	0	0	0	3	3	0	0	13	13	0	0	0	0	0	0	0	0	0	0	5	5
RL5A-G4	GCGTCCAAGTTGACGTAAGACCAT	56	56	0	0	0	0	0	0	0	0	0	0	0	0	0	0	0	0	0	0	0	0	0	0	0	0
RL5A-G5	TCCCATCTTTTCCACTCGGTGGAA	0	0	0	0	0	0	0	0	0	0	0	0	0	0	0	0	0	0	0	0	0	0	0	0	0	0
RL5A-G6	TCCCATCTTTTCCATTCAGTGGAA	0	0	0	0	0	0	0	0	1259	1259	0	0	0	0	1136	1136	1	1	300	294	0	0	0	0	0	0
RL6-G1	CGCATCCACATATATCAACGTCAT	1	1	2	2	0	0	0	0	677	677	0	0	0	0	0	0	0	0	0	0	0	0	74	74	0	0
RL6-G2	CAGATGGCTCCATTTCGTAGTCAT	1	1	0	0	0	0	0	0	0	0	80	83	0	0	102	102	0	0	970	970	490	489	428	429	2043	2043
RL6-G3	GTATCCACTTGTTCAGGACCGTTA	0	0	162	162	5348	5350	0	0	0	0	0	0	0	0	317	317	0	0	1383	1383	6	6	59	59	0	0
RL6-G4	CGTTGCCGTCATATTGCATATGGT	0	0	0	0	0	0	0	0	0	0	0	0	0	0	1	1	0	0	843	842	1375	1376	659	658	0	0
RL6-G5	GTATCCACTTGAATGTTGCC	0	0	0	0	0	0	0	0	0	0	0	0	0	0	0	0	0	0	0	0	0	0	0	0	4	4
RL6-G6	CCAATGGCTCCATCTAGTAGTCAT	0	0	0	0	0	0	0	0	0	0	0	0	0	0	0	0	0	0	0	0	0	0	0	0	0	0
RL6-G7	TATTGACGTCATATGTATGATTAC	232	232	0	0	0	0	0	0	0	0	0	0	0	0	0	0	0	0	0	0	0	0	0	0	0	0
RL12-G1A	AATGCAAATGAAAACGTCACTACA	0	0	0	0	0	0	1179	1179	0	0	0	0	0	0	0	0	0	0	0	0	0	0	0	0	0	0
RL12-G1B	TCATGGTAAATGTAACTGAAAACG	0	0	0	0	0	0	0	0	0	0	0	0	0	0	0	0	0	0	397	397	972	972	753	753	0	0
RL12-G2	CAATGGTAACAAATACAACTCAAA	1	1	0	0	0	0	0	0	0	0	0	0	1	1	220	220	0	0	0	0	0	0	0	0	1	1
RL12-G3	TAATAAATACAACTGAAAACAGTA	0	0	0	0	0	0	0	0	0	0	0	0	0	0	0	0	0	0	271	270	0	0	0	0	0	0
RL12-G4A	AATACCTCATTTATGAGCACGCCA	177	177	0	0	0	0	1	1	0	0	0	0	0	0	2	2	0	0	0	0	0	0	61	61	753	753
RL12-G4B	CTTCGCACAACGTATCCACTAATA	1	1	4	4	0	0	0	0	0	0	0	0	0	0	1178	1178	0	0	194	194	2	2	0	0	0	0
RL12-G5	CTATGGATAACACTACGTCCAACC	0	0	0	0	0	0	0	0	0	0	0	0	0	0	0	0	0	0	0	0	0	0	0	0	0	0
RL12-G6	TAATCTAACCACACATGGAGATTA	0	0	0	0	0	0	0	0	0	0	0	0	0	0	0	0	0	0	705	684	514	514	501	501	0	0
RL12-G7	CAGTTTACAACACCACGTACATTC	0	0	0	0	0	0	0	0	0	0	0	0	0	0	0	0	0	0	79	79	0	0	0	0	0	0
RL12-G8	CCATTCATAACACTACGTACAGTC	0	0	0	0	5856	5854	6	6	0	0	0	0	0	0	0	0	0	0	2175	2171	0	0	0	0	0	0
RL12-G9	ACAGCACCGTGTTTACTATGAGCA	0	0	60	60	0	0	0	0	0	0	0	0	0	0	0	0	0	0	0	0	0	0	0	0	0	0
RL12-G10	CAGACATGACCGCAACAATTATTA	0	0	0	0	0	0	0	0	657	657	16	16	0	0	0	0	0	0	0	0	0	0	33	33	0	0
RL13-G1	ATCAGCAACCACTTCAACGCTTTC	0	0	1	1	0	0	0	0	0	0	0	0	0	0	0	0	0	0	775	777	542	543	751	754	0	0
RL13-G2	GCGAATATTAACAACGTTCTCTAC	1	1	0	0	0	0	0	0	0	0	0	0	4	4	2564	2564	0	0	0	0	0	0	2	2	3	3
RL13-G3	AGCATTCATACCACTCCCTGGTTG	31	31	0	0	0	0	0	0	0	0	0	0	0	0	0	0	0	0	0	0	0	0	0	0	0	0
RL13-G4A	TACAAAGTCGTCGGCAATAACCAC	126	126	1	1	0	0	0	0	0	0	0	0	0	0	0	0	0	0	0	0	0	0	0	0	3314	3314
RL13-G4B	TACAAAACCGTCGAAAACAACCAC	0	0	1	1	0	0	0	0	0	0	0	0	0	0	1	1	0	0	0	0	3	3	0	0	0	0
RL13-G5	AAAAGGCACGCAAATATCCACAAG	0	0	0	0	0	0	0	0	0	0	0	0	0	0	0	0	0	0	0	0	0	0	0	0	0	0
RL13-G6	AACATGCGGTCGTAGCTACGAAGT	0	0	0	0	0	0	0	0	0	0	0	0	9519	9519	0	0	1	1	1117	1117	230	230	260	260	4962	4962
RL13-G7	CAGCTTCATCAACCATACTATCTC	0	0	0	0	0	0	0	0	0	0	0	0	0	0	0	0	0	0	24	24	0	0	0	0	0	0
RL13-G8	CAGCGTCGTCAACCATAATATCTC	0	0	0	0	5059	5041	800	800	0	0	0	0	0	0	0	0	0	0	2458	2458	0	0	0	0	0	0
RL13-G9	AACCATCCATATATCCACAAAATC	0	0	0	0	0	0	0	0	0	0	0	0	0	0	0	0	0	0	117	117	0	0	0	0	0	0
RL13-G10	AACAAAACCGTTGATAGCAACCAC	0	0	0	0	0	0	0	0	0	0	19	18	0	0	0	0	0	0	0	0	63	63	258	258	0	0
UL1-G1	ATTCCAGTTTCTGGAACTACGGCA	0	0	0	0	0	0	0	0	0	0	0	0	0	0	0	0	0	0	1198	1199	610	610	1263	1264	0	0
UL1-G2	ATTCCAGCTTCTGGAACTATGGCG	2	2	0	0	0	0	0	0	3	3	0	0	2	2	577	577	1	1	0	0	0	0	1	1	6	6
UL1-G3	TCAACATATAGAAACACTCATTAC	0	0	0	0	0	0	0	0	0	0	0	0	0	0	0	0	0	0	142	142	0	0	0	0	0	0
UL1-G4	CAATAATCCCTGTAATTATTCCAG	125	125	2	2	0	0	1	1	0	0	1	1	0	0	2386	2386	0	0	94	94	66	66	0	0	0	0
UL1-G5	TGACAATCCCTGCAATTATCCAG	0	0	0	0	0	0	0	0	0	0	0	0	0	0	0	0	0	0	0	0	0	0	0	0	0	0
UL1-G6	GTAATTCAGGGAGATACAGTTTAT	0	0	0	0	0	0	0	0	0	0	1	1	7033	7033	0	0	1	1	533	533	459	459	472	472	5275	5275
UL1-G7	TGCGGTGGACACCGTTTTCTTT	0	0	0	0	0	0	0	0	0	0	0	0	0	0	0	0	1	1	26	26	0	0	0	0	0	0
UL1-G8	TGCGGTTGGACACCATTTTACTCG	31	31	0	0	4778	4759	0	0	0	0	0	0	0	0	0	0	0	0	3055	3055	0	0	0	0	0	0
UL1-G9	TACAAATACCCATCACAGTAGCTG	0	0	0	0	5	2	0	0	0	0	0	0	0	0	0	0	4	4	3	3	0	0	0	0	0	0
UL1-G10	CTAATTCAGGAAGACACAGTTTAT	0	0	0	0	0	0	0	0	0	0	53	53	0	0	0	0	0	0	0	0	0	0	4	4	0	0
UL9-G1	CCTGTACACAATTCATTAACAATA	131	129	0	0	0	0	0	0	2	2	0	0	0	0	1	1	3	3	0	0	30	30	6	6	5	5
UL9-G2	ACAACATACAGTAGAAACTATTAT	0	0	0	0	0	0	0	0	0	0	0	0	0	0	1247	1247	0	0	370	371	0	0	0	0	0	0
UL9-G3	CCCACACAAAAGTCACCCAAAACA	3	3	1	1	0	0	0	0	0	0	0	0	0	0	731	731	60	60	1	1	1	1	0	0	0	0
UL9-G4	ACTACATATAGTAGAAATTACTAT	0	0	0	0	5724	5723	0	0	0	0	0	0	0	0	0	0	0	0	2229	2227	669	667	634	634	0	0
UL9-G5	CCCGTACGCAGCTCATACACAACA	0	0	0	0	0	0	0	0	0	0	0	0	0	0	0	0	0	0	0	0	0	0	0	0	0	0
UL9-G6	TCCACACGTAATTCATTAACGATA	3	3	0	0	0	0	273	273	0	0	0	0	0	0	0	0	0	0	214	214	972	972	229	229	2273	2273
UL9-G7	CCCACACATAATCTATTAACAGCA	0	0	0	0	0	0	0	0	0	0	0	0	52	52	0	0	0	0	1006	1006	0	0	87	87	0	0
UL9-G8	CCACACATAATTCATTAACAATGT	0	0	0	0	0	0	0	0	0	0	25	25	0	0	0	0	0	0	0	0	0	0	33	33	0	0
UL9-G9	TCCACATATAATTCATTAACTATA	1	1	42	42	0	0	0	0	1127	1127	0	0	0	0	2	2	0	0	303	303	6	6	0	0	1382	1382
UL11-G1	CGATGGACTTTAGACACATGTTAC	333	333	0	0	5142	5110	0	0	0	0	0	0	0	0	0	0	0	0	2934	2934	986	986	1159	1158	2487	2487
UL11-G2	CACATTATCATAAACCTCCCGGAT	0	0	0	0	0	0	0	0	0	0	0	0	0	0	0	0	0	0	0	0	0	0	0	0	8	8
UL11-G3	AATCAACATCACCATAAATTTCCC	0	0	0	0	0	0	0	0	1	1	0	0	0	0	0	0	1	1	570	570	0	0	0	0	6	6
UL11-G4	CAATTGGCTTCACAGTCGACATCA	2	2	0	0	0	0	1884	1884	0	0	0	0	0	0	0	0	0	0	0	0	684	684	1059	1061	0	0
UL11-G5	AGTACGCATCGCCATAGAGCTCCC	0	0	0	0	0	0	0	0	0	0	0	0	0	0	601	601	0	0	739	739	0	0	0	0	0	0
UL11-G6	TCAATGTAACGGAAGAATATACTG	0	0	1	1	0	0	0	0	0	0	0	0	0	0	1439	1439	0	0	38	38	4	4	0	0	0	0
UL11-G7	GACCGCGTTCGTACACCAATCCCA	0	0	0	0	0	0	0	0	0	0	32	32	0	0	0	0	0	0	0	0	19	26	51	51	0	0
UL73-G1	AACCTCTGCAACTACATCAAAGTC	0	0	1	1	0	0	0	0	0	0	0	0	0	0	0	0	0	0	320	320	90	90	26	26	0	0
UL73-G2	CCAAGTCCTTCTAGTGTGTCGACG	0	0	0	0	0	0	0	0	0	0	0	0	0	0	330	330	0	0	28	26	506	506	163	163	0	0
UL73-G3A	AATCGTCCAGTTCTAGCGTGTCAA	0	0	0	0	4888	4853	0	0	0	0	82	82	0	0	268	268	0	0	1105	1105	57	57	24	24	0	0
UL73-G3B	CCGAGTCCTCCTAGTTCTAGTGTA	0	0	0	0	0	0	0	0	0	0	0	0	0	0	0	0	0	0	0	0	0	0	0	0	0	0
UL73-G4A	ACAACTGCAACAACTTCTACAACA	266	266	0	0	0	0	0	0	0	0	0	0	0	0	0	0	0	0	1636	1636	935	934	1016	1013	497	497
UL73-G4B	AGTCCCTCTAGCTCTACTCACACC	0	0	0	0	39	39	0	0	0	0	0	0	0	0	0	0	0	0	168	168	54	54	6	6	3	3
UL73-G4C	AGTCCTCTAGCTCTACTCGCACC	0	0	0	0	0	0	0	0	1	1	0	0	0	0	0	0	0	0	1	1	0	0	0	0	3	3
UL73-G4D	CGTCCCTCTAGTTCTACTCACGCC	0	0	0	0	0	0	1331	1331	0	0	1	1	0	0	15	15	0	0	0	0	0	0	0	0	0	0
UL74-G1A	GTACCAAGCCCCTCCAAGGACGAT	0	0	157	157	0	0	2	2	0	0	1	1	1560	1560	0	0	1	1	487	487	49	49	10	10	794	794
UL74-G1B	GTAGACAGTACTTTACCTGACCAG	0	0	0	0	5360	5321	0	0	0	0	41	41	0	0	492	492	45	45	1529	1530	0	0	110	110	0	0
UL74-G1C	GTACTGTACCTAACCAGTAACGTC	0	0	0	0	0	0	0	0	0	0	0	0	0	0	0	0	0	0	1	1	0	0	0	0	4	4
UL74-G2A	CCATATCCTAGGACGAAATGGTGC	0	0	0	0	0	0	0	0	0	0	0	0	0	0	0	0	0	0	42	42	2	2	0	0	0	0
UL74-G2B	GTACTTTACCTATCCAGGGACGTC	0	0	0	0	0	0	0	0	0	0	0	0	0	0	477	477	0	0	51	51	732	732	233	233	0	0
UL74-G3	TTTGATAATACCCTGCCATAGGGA	129	129	1	1	0	0	0	0	0	0	0	0	0	0	0	0	0	0	581	580	1597	1597	771	765	163	163
UL74-G4	TGACTCTTTACTCGAAAACGCGCT	0	0	0	0	0	0	0	0	0	0	0	0	0	0	0	0	0	0	559	559	137	137	0	0	0	0
UL74-G5	TAAGATAGCACGGTATACGGCCAG	0	0	0	0	0	0	0	0	0	0	0	0	0	0	0	0	0	0	0	0	0	0	0	0	0	0
UL120-G1A	TATTGTGTGTTCTTTGATGTACTG	2	2	0	0	0	0	989	989	0	0	1	1	0	0	0	0	0	0	1736	1715	0	0	0	0	0	0
UL120-G1B	ATATTGTGTGTTTTTGGAGTACT	0	0	0	0	0	0	0	0	0	0	0	0	0	0	0	0	0	0	0	0	0	0	0	0	0	0
UL120-G2A	TGGTGAGAAAGCGTGTTTTCAACG	1	1	0	0	0	0	0	0	20	20	57	57	1	1	0	0	1	1	0	0	33	33	0	0	1198	1198
UL120-G2B	AAACTGTGTGTTCTGTGGAATACT	0	0	0	0	4400	4403	0	0	0	0	0	0	0	0	1	1	0	0	0	0	1098	1098	634	635	0	0
UL120-G3A	CCGTGTATCCCGTGGAATACTGAT	8	8	0	0	0	0	0	0	0	0	0	0	0	0	0	0	736	736	63	63	553	553	556	556	0	0
UL120-G3B	TCGTGTGTTATGTGGAATACTGAT	0	0	0	0	0	0	0	0	0	0	0	0	0	0	38	38	0	0	0	0	0	0	0	0	0	0
UL120-G4A	GTACGATGCGGAAGGACGATCATC	0	0	18	18	0	0	0	0	0	0	0	0	0	0	0	0	0	0	27	27	0	0	0	0	0	0
UL120-G4B	TGTGTACGGTGCGGGAGAACGATC	107	107	0	0	110	110	1473	1473	0	0	0	0	0	0	1182	1182	0	0	933	933	3	8	39	39	835	835
UL146-G1	TTCGTTATGTTAACATTATACCAC	0	0	0	0	0	0	187	187	0	0	0	0	0	0	0	0	215	215	0	0	0	0	0	0	0	0
UL146-G2	TTATTCCTTCAGACCTACTAGGGT	2	2	0	0	0	0	0	0	0	0	0	0	0	0	834	834	1302	1302	0	0	0	0	0	0	0	0
UL146-G3	TTATTGTTTGACAGCTATTAAGGT	13	13	0	0	0	0	0	0	0	0	0	0	0	0	0	0	0	0	671	671	0	0	0	0	0	0
UL146-G4	CCACTAGAAAAGGTAGGCTTAGTA	0	0	0	0	0	0	0	0	0	0	0	0	0	0	0	0	0	0	0	0	0	0	0	0	0	0
UL146-G5	CGAGTATTCGATCTTTGTACTACT	0	0	0	0	0	0	0	0	0	0	0	0	0	0	0	0	0	0	0	0	0	0	0	0	0	0
UL146-G6	TTAACTTGGTGGAGTAAACGATTT	0	0	0	0	0	0	0	0	0	0	0	0	0	0	0	0	0	0	0	0	0	0	0	0	0	0
UL146-G7	TTATTTCCAACGCGGACGTTTGTT	0	0	0	0	0	0	0	0	0	0	0	0	0	0	1	1	0	0	302	302	443	445	376	375	0	0
UL146-G8	AGTCGCCAAGGCGGGTGTCTATTG	0	0	0	0	0	0	0	0	0	0	0	0	0	0	0	0	89	89	0	0	0	0	0	0	996	996
UL146-G9	TTAAAATTTTAGTTTCCAAGGCGG	0	0	0	0	0	0	0	0	0	0	0	0	0	0	0	0	0	0	2583	2584	1240	1240	894	894	0	0
UL146-G10	TAATTTCAGTCCTTTTTTCTACTT	0	0	0	0	0	0	0	0	0	0	0	0	0	0	0	0	0	0	0	0	0	0	0	0	0	0
UL146-G11	ATCCAACCATTTTGATAAATGGTG	28	28	0	0	0	0	0	0	0	0	0	0	0	0	0	0	0	0	16	17	0	0	0	0	0	0
UL146-G12	CCCAAGCATACGGTCTTGCCTTGG	0	0	0	0	4823	4797	0	0	0	0	0	0	0	0	1468	1468	0	0	578	578	40	40	53	53	0	0
UL146-G13	CTAAATCATTATATTTTTTCGACC	188	188	0	0	19	19	0	0	0	0	0	0	0	0	0	0	0	0	0	0	2	2	3	3	0	0
UL146-G14	TTATAATTTCCATTTTGGACGTTT	0	0	0	0	0	0	838	838	0	0	0	0	0	0	0	0	0	0	0	0	0	0	8	8	753	195
UL139-G1A	GCGCGAGCTGTGGCGCCCCCAAAT	0	0	0	0	0	0	0	0	0	0	0	0	0	0	0	0	0	0	2676	2649	2	2	0	0	0	0
UL139-G1B	GAGCTGTGATACCTCCCAAGTACT	0	0	0	0	0	0	0	0	0	0	0	0	0	0	0	0	0	0	583	583	0	0	0	0	0	0
UL139-G2	AAGACGAAGTAGATTGACTGGAAT	0	0	0	0	0	0	0	0	0	0	0	0	0	0	0	0	270	270	0	0	29	29	0	0	1	1
UL139-G3	AATTATTGCCGCCAAAACCATTAA	0	0	172	172	4738	4715	0	0	246	246	0	0	0	0	1718	1718	0	0	282	282	5	5	84	84	0	0
UL139-G4	ACTGCCGTTAGAGGCATTAATACA	3	3	1	1	0	0	998	998	3	3	69	69	2	2	0	0	8	8	1	1	53	53	15	15	1134	1134
UL139-G5	ACCAGGTACCGTTTGTGCCATCGA	114	114	0	0	0	0	0	0	0	0	0	0	0	0	0	0	633	633	78	78	1222	1221	1662	1661	0	0
UL139-G6	AATTACTGCCGCCAAAAGCTTGAA	0	0	0	0	0	0	0	0	0	0	0	0	0	0	0	0	0	0	0	0	0	0	0	0	0	0
UL139-G7	AATTACTGCCACCAAAGGCTTGAA	0	0	0	0	0	0	398	398	309	309	0	0	0	0	0	0	0	0	845	846	470	470	660	660	223	223
UL139-G8	GAGTTGTGGGACTGTCCAATTACT	1	1	0	0	0	0	0	0	0	0	0	0	0	0	840	840	0	0	0	0	1	1	0	0	0	0
RL5A-G1-D1	TGTACCATTATTAGTCACCACCGT	0	0	0	0	86	86	0	0	0	0	0	0	4839	4839	0	0	0	0	0	0	0	0	0	0	0	0
RL5A-G1-D2-1	GAAACACAGACCGAGCGTTGTACC	0	0	0	0	8	8	4	4	0	0	0	0	0	0	0	0	1743	1743	5	5	0	0	0	0	0	0
RL5A-G1-D2-2	GAAACACAGACCGCGCGTTGTACC	0	0	0	0	6245	6240	1203	1203	0	0	0	0	0	0	0	0	8	8	1635	1635	0	0	0	0	0	0
UL111A-D1	ATCGAGTAAAACGGTCTTTCTTTT	0	0	0	0	0	0	0	0	0	0	0	0	0	0	0	0	0	0	0	0	0	0	0	0	0	0
UL111A-WT	ATCGAGTAAAACCTACGTTGGTAG	148	148	0	0	3695	3699	1	1	94	94	0	0	1	1	2033	2033	1	1	3601	3585	1616	1617	1669	1669	2612	2612
US9-D1-1	TTAGCCTCTTCCTCGAACCCAGGG	176	179	83	83	2791	2788	1078	1079	0	0	46	45	0	0	434	434	0	0	405	404	857	857	2604	2608	2	2
US9-D1-2	TTAGCCTCTTCCTTGAACCCAGGG	0	0	0	0	1	1	0	0	0	0	0	0	0	0	741	741	0	0	2045	2045	0	0	0	0	0	0
US9-WT	TCAATCGTCTTTAGCCTCTTCTTC	0	0	1	1	3	3	0	0	0	0	2	2	0	0	2	2	1	1	0	0	0	0	1	1	1	1

SUMMARY DATA[a]																															
Donor		243						248		258		259				264		277		278						281		283		288	
HIV status		Positive						Positive		Positive		Positive				Positive		Positive		Positive						Positive		Positive		Positive	
Breast sample		Left		Right		Right		Right		Right		Left		Right		Left		Right		Left		Right		Right		Right		Right		Right	
Time postpartum (weeks)		16		4		16		4		4		16		16		16		16		16		4		16		4		16		4	
HCMV load (genome equivalents/ml)		643,895		65,511,020		795,092		441,679		610,613		2,366,193		5,053,047		388,519		250,377		3,751,776		294,246,272		4,370,800		20,291,530		274,391		31,574,022	
Genome equivalent copies for library (no.)		3,219		98,267		3,975		2,208		6,106		3,549		5,053		3,885		2,504		3,752		588,493		4,371		40,583		2,744		47,361	
Pre-enrichment PCR cycles (no.)		18		18		18		18		12		18		18		12		12		18		18		18		18		12		18	
Post-enrichment PCR cycles (no.)		16		13		16		16		16		16		16		19		16		13		16		13		19		19		19	
Dataset name		243L16		243R4		243R16		248R4		258R4		259L16		259R16		264L16		277R16		278L16		278R4		278R16		281R4		283R16		288R4	
Original reads (no.)		2,264,444		2,946,096		1,772,662		1,708,660		2,921,988		3,240,060		2,903,026		2,578,802		2,918,986		2,960,564		3,505,608		2,966,644		5,437,046		2,720,998		2,973,632	
Trimmed reads (no.)		2,264,420		2,946,068		1,772,622		1,708,648		2,921,930		3,239,952		2,903,006		2,578,790		2,918,964		2,960,512		3,505,526		2,966,610		5,436,914		2,720,992		2,973,542	
Purged reads (no.)		2,205,834		2,846,886		1,538,228		1,550,818		1,786,824		3,054,566		2,743,430		2,529,902		2,828,574		2,941,048		3,010,816		2,948,822		3,297,974		2,472,672		2,298,302	
ENA purged short read accession no.		ERR3163235		ERR3163236		ERR3163251		ERR3163252		ERR3163253		ERR3163254		ERR3163257		ERR3163258		ERR3163259		ERR3163274		ERR3163275		ERR3163276		ERR3163277		ERR3163329		ERR3163330	
GenBank complete genome accession no.				MK422176				MK290743																				MK290744			
Strains (no.)		2		1		1		1		2		3		3		2		2		2		2		2		2		1		3	
ASSEMBLY DATA (STRAIN MERLIN REFERENCE)[b]		Trimmed	Purged	Trimmed	Purged	Trimmed	Purged	Trimmed	Purged	Trimmed	Purged	Trimmed	Purged	Trimmed	Purged	Trimmed	Purged	Trimmed	Purged	Trimmed	Purged	Trimmed	Purged	Trimmed	Purged	Trimmed	Purged	Trimmed	Purged	Trimmed	Purged
HCMV reads (no.)		1,915,924	1,915,917	2,460,275	2,460,244	1,376,422	1,376,406	1,419,252	1,419,250	1,575,357	1,575,339	2,698,781	2,698,699	2,413,399	2,413,367	2,257,987	2,257,661	2,535,388	2,535,043	2,582,018	2,581,976	2,633,385	2,633,328	2,582,325	2,582,300	2,903,965	2,903,932	2,194,148	2,193,734	1,993,068	1,992,977
HCMV reads (%) (1)		85	77	84	77	78	77	83	77	54	77	83	77	83	77	88	77	87	77	87	77	75	77	87	77	53	77	81	77	67	77
Coverage extent (% genome) (2)		98	98	96	96	97	97	97	97	97	97	98	98	98	98	98	98	98	98	97	97	97	97	97	97	97	97	97	97	98	98
Coverage depth (total fragment reads/nt) (3)		2,029	2,029	3,040	3,040	1,453	1,453	1,503	1,503	1,967	1,967	3,362	3,362	3,015	3,015	2,818	2,817	3,157	3,156	3,228	3,228	3,246	3,246	3,233	3,233	3,458	3,458	2,761	2,760	2,484	2,484
Coverage depth (unique fragment reads/nt) (4)		19	19	211	211	122	122	47	47	79	79	123	123	119	119	77	77	178	178	382	382	243	243	553	553	125	125	57	57	48	48
GENOTYPING DATA (SHORT MOTIF-MATCHING COUNTS)[c]		Trimmed	Purged	Trimmed	Purged	Trimmed	Purged	Trimmed	Purged	Trimmed	Purged	Trimmed	Purged	Trimmed	Purged	Trimmed	Purged	Trimmed	Purged	Trimmed	Purged	Trimmed	Purged	Trimmed	Purged	Trimmed	Purged	Trimmed	Purged	Trimmed	Purged
UL73-G1-5'	GCGTATCAACTACC	0	0	0	0	0	0	0	0	99	100	755	754	399	399	1	0	1749	1738	0	1	0	0	0	0	0	0	0	1	314	314
UL73-G2-5'	GTGTGTCGACGAGT	0	0	3	3	0	0	0	0	1	2	1364	1364	566	552	1	0	0	1	1	1	4	4	1	2	0	0	0	0	0	0
UL73-G3A-5'	GCGTGTCAACAAGC	139	140	3	2	0	0	1	1	1798	1798	42	41	2	2	532	531	0	0	3222	3229	2378	2382	3089	3090	175	176	2921	2903	10	10
UL73-G3B-5'	GTGTATCAACGGTA	7	7	0	0	0	0	1558	1553	0	0	1	1	0	0	0	0	0	0	0	0	2	1	0	0	3	4	196	196	3	0
UL73-G4A-5'	GCACCTTAACAACC	0	1	1	1	2	0	0	1	334	331	3	2	2	1	0	0	3787	3788	1	1	0	2	3	2	0	0	0	0	108	107
UL73-G4B-5'	ACACCTCAACGACC	2049	2049	3123	3121	2077	2077	0	0	21	21	1526	1526	1528	1527	4559	4557	0	1	3	4	2	2	3	4	3	2	0	0	185	185
UL73-G4C-5'	GCACCTCAACAACC	0	1	1	2	1	0	2	1	19	17	5	5	2	3	0	2	6	5	5	3	2	2	5	4	5	2	1	1	4	2
UL73-G4D-5'	ACGCCTCAACAACC	9	8	14	13	3	2	2	2	5	5	19	20	19	17	7	7	15	13	823	819	932	931	570	572	3422	3423	9	15	2227	2226
UL74-G1A-5'	AAACGACWATTT	16	15	49	45	28	25	8	4	44	30	344	343	363	364	23	19	861	860	27	22	38	27	16	16	46	15	18	12	87	78
UL74-G1B-5'	AAAAGGATATCT	11	4	31	23	4	0	2	0	2292	2233	17	11	30	20	315	315	125	120	3127	3125	2188	2170	3308	3308	347	248	2755	2741	54	13
UL74-G1C-5'	AAAGGGAACCTT	7	2	5	1	9	2	1	0	48	1	18	12	13	6	1	1	11	5	6	7	36	3	6	6	66	3	5	1	31	4
UL74-G2A-5'	AACCTATTCCTT	5	7	13	7	4	0	619	614	70	2	11	6	21	6	7	5	10	1	4	4	37	7	6	2	82	8	19	3	27	3
UL74-G2B-5'	AGAGCGACATAT	0	0	8	8	0	0	2	2	3	1	1064	1065	873	871	4	4	1	1	9	8	14	8	5	5	21	16	3	2	9	5
UL74-G3-5'	CGAGCCAGGATT	2	4	3	2	9	2	5	3	115	95	16	6	5	2	2	2	2852	2850	5	7	13	2	4	6	52	0	8	4	62	43
UL74-G4-5'	CGACAGGTGATT	28	31	21	8	8	12	17	16	6	4	13	15	24	23	18	28	10	8	14	11	17	8	9	10	18	9	7	7	11	6
UL74-G5-5'	TGTCTACATCAT	14	10	34	31	5	3	7	4	37	4	33	24	38	33	3	5	59	58	456	454	631	607	479	477	2149	2080	17	5	1792	1765
UL74-G1A-C	CCTTGTGGTACTG	2	2	3	5	1	0	3	3	8	1	399	396	382	381	1	1	1348	1350	3	3	8	4	11	9	16	5	3	2	54	53
UL74-G1B-C	TCTTGCGGTACGG	1	1	12	11	5	5	2	2	2362	2359	15	13	43	44	809	809	79	78	2559	2560	1927	1929	2906	2907	392	390	3008	3008	3	3
UL74-G1C-C	TCTTGTGGTACAG	1	1	6	5	2	1	6	3	6	1	5	4	6	7	10	9	4	4	4	4	4	2	5	4	12	6	1	1	5	4
UL74-G2A-C	TCGTGTGGCGCAG	6	7	14	19	0	0	1162	1161	4	4	12	11	11	13	6	7	3	5	17	17	8	10	11	10	13	10	2	6	12	8
UL74-G2B-C	CCTTGCGGTACAG	7	3	6	3	2	0	4	0	11	4	1104	1083	1090	1091	11	10	13	8	2	2	6	3	6	7	126	122	5	2	4	3
UL74-G3-C	TCTTGTGGCACTG	6	5	31	30	8	5	8	0	319	302	7	5	14	9	2	2	3410	3408	26	26	25	21	39	38	55	18	6	6	206	191
UL74-G4-C	TCCTGTGGYACGA	2826	2826	3310	3309	1430	1427	3	3	12	3	1493	1478	1741	1735	2883	2881	8	6	7	7	13	7	4	7	17	11	6	6	17	13
UL74-G5-C	CCTTGYGGCACAG	3	4	13	8	12	11	12	8	17	11	37	22	20	15	4	3	9	15	547	547	465	449	461	460	2025	1984	26	17	1867	1858
UL74-G1A-3'	TATTACTACCGCC	3	0	3	3	4	3	0	0	4	3	434	434	250	251	4	5	1657	1658	5	5	3	2	0	4	3	1	2	1	3	4
UL74-G1B-3'	TGTTACTACCACC	6	5	18	15	15	15	3	1	2364	2356	15	12	17	17	672	667	20	20	3169	3168	2001	1998	3109	3109	240	209	5077	5072	18	13
UL74-G1C-3'	GGTTACCACCAGC	1	2	9	9	5	4	5	6	7	1	12	10	7	7	9	9	6	3	45	39	47	45	37	34	21	19	2	1	6	4
UL74-G2A-3'	TGTTACCACCACC	9	9	53	53	17	9	1206	1206	63	29	87	78	70	73	27	23	35	33	58	60	57	43	71	67	82	43	55	48	51	31
UL74-G2B-3'	TGTTACAACCACC	4	3	14	9	7	4	6	7	12	4	1615	1615	1460	1462	11	9	68	68	16	16	11	10	17	13	35	16	8	10	19	11
UL74-G3-3'	TGCTACCACCACT	6	2	7	7	3	1	4	2	416	397	66	65	13	13	2	4	3385	3381	6	7	22	11	7	8	56	5	26	11	16	7
UL74-G4-3'	TCCTATTGTCCCA	3936	3936	3171	3173	974	972	2	1	2	0	1482	1481	1660	1655	3103	3107	1	0	0	0	7	3	3	3	14	1	1	0	340	330
UL74-G5-3'	TGCTACCGCTGCT	17	16	47	45	20	5	17	5	53	7	66	52	43	42	11	10	18	11	643	644	541	534	395	392	4254	4086	40	23	2251	2178
GENOTYPING DATA (LONG MOTIF-MATCHING COUNTS)[c]		Trimmed	Purged	Trimmed	Purged	Trimmed	Purged	Trimmed	Purged	Trimmed	Purged	Trimmed	Purged	Trimmed	Purged	Trimmed	Purged	Trimmed	Purged	Trimmed	Purged	Trimmed	Purged	Trimmed	Purged	Trimmed	Purged	Trimmed	Purged	Trimmed	Purged
RL5A-G1	ATTTTATCATTAAACCCCAATATT	4173	4173	3188	3190	2167	2167	1811	1811	0	0	2281	2281	3242	3242	889	889	4210	4211	0	0	0	0	1	1	166	166	0	0	320	320
RL5A-G2	ATGTCCACGTTGAATTCAACTTAG	0	0	1	1	0	0	0	0	0	0	2223	2226	970	970	1	1	0	0	633	633	1042	1047	333	333	1	1	3	3	2816	2816
RL5A-G3	GCTTCCAAGTTGACGTAAGATCGT	0	0	0	0	0	0	0	0	0	0	0	0	0	0	0	0	0	0	3	3	0	0	0	0	0	0	0	0	0	0
RL5A-G4	GCGTCCAAGTTGACGTAAGACCAT	0	0	0	0	0	0	0	0	0	0	0	0	0	0	0	0	0	0	0	0	0	0	0	0	0	0	0	0	0	0
RL5A-G5	TCCCATCTTTTCCACTCGGTGGAA	0	0	0	0	0	0	0	0	0	0	0	0	0	0	0	0	0	0	0	0	0	0	0	0	0	0	0	0	0	0
RL5A-G6	TCCCATCTTTTCCATTCAGTGGAA	0	0	0	0	0	0	0	0	2048	2043	0	0	1	1	0	0	0	0	2766	2767	3144	3150	2630	2633	2755	2754	0	0	3	3
RL6-G1	CGCATCCACATATATCAACGTCAT	4	4	0	0	2	2	1528	1528	0	0	6	6	0	0	706	707	0	0	2	2	1	1	0	0	3	3	2	2	3	3
RL6-G2	CAGATGGCTCCATTTCGTAGTCAT	0	0	0	0	0	0	0	0	0	0	445	445	227	226	3272	3267	0	0	0	0	0	0	0	0	0	0	0	0	0	0
RL6-G3	GTATCCACTTGTTCAGGACCGTTA	0	0	29	29	17	17	0	0	2864	2863	2032	2034	2290	2290	0	0	5261	5261	3255	3249	2765	2768	3107	3106	4392	4392	14	14	243	243
RL6-G4	CGTTGCCGTCATATTGCATATGGT	0	0	0	0	0	0	0	0	2	2	1745	1745	931	931	0	0	0	0	867	867	930	930	505	505	0	0	5122	5124	1268	1264
RL6-G5	GTATCCACTTGAATGTTGCC	0	0	0	0	0	0	0	0	0	0	0	0	0	0	0	0	0	0	0	0	0	0	0	0	0	0	0	0	0	0
RL6-G6	CCAATGGCTCCATCTAGTAGTCAT	2666	2666	3072	3072	1288	1289	0	0	0	0	0	0	0	0	0	0	0	0	1	1	0	0	0	0	0	0	0	0	0	0
RL6-G7	TATTGACGTCATATGTATGATTAC	0	0	0	0	0	0	0	0	0	0	0	0	0	0	0	0	0	0	0	0	0	0	0	0	0	0	0	0	0	0
RL12-G1A	AATGCAAATGAAAACGTCACTACA	0	0	0	0	0	0	0	0	0	0	1745	1745	1445	1445	0	0	0	0	702	702	1841	1838	603	603	3087	3085	0	0	1	1
RL12-G1B	TCATGGTAAATGTAACTGAAAACG	0	0	0	0	0	0	0	0	0	0	0	0	0	0	0	0	0	0	0	0	0	0	0	0	0	0	0	0	0	0
RL12-G2	CAATGGTAACAAATACAACTCAAA	984	984	2666	2666	467	467	0	0	0	0	0	0	0	0	0	0	0	0	1	1	0	0	0	0	0	0	0	0	0	0
RL12-G3	TAATAAATACAACTGAAAACAGTA	0	0	0	0	0	0	0	0	2423	2422	0	0	0	0	0	0	0	0	0	0	0	0	0	0	0	0	0	0	0	0
RL12-G4A	AATACCTCATTTATGAGCACGCCA	0	0	27	27	0	0	2462	2463	0	0	0	0	0	0	0	0	3298	3292	0	0	0	0	0	0	0	0	0	0	0	0
RL12-G4B	CTTCGCACAACGTATCCACTAATA	0	0	0	0	0	0	0	0	0	0	0	0	0	0	0	0	0	0	0	0	0	0	0	0	0	0	3878	3878	0	0
RL12-G5	CTATGGATAACACTACGTCCAACC	0	0	0	0	0	0	0	0	0	0	0	0	0	0	0	0	0	0	0	0	0	0	0	0	0	0	0	0	0	0
RL12-G6	TAATCTAACCACACATGGAGATTA	0	0	135	135	2	2	0	0	0	0	447	447	559	559	0	0	1296	1296	0	0	0	0	0	0	269	269	2	2	3062	3062
RL12-G7	CAGTTTACAACACCACGTACATTC	0	0	0	0	0	0	0	0	0	0	0	0	0	0	0	0	0	0	0	0	0	0	0	0	4	4	0	0	589	589
RL12-G8	CCATTCATAACACTACGTACAGTC	0	0	0	0	0	0	0	0	0	0	3467	3467	2655	2654	420	420	0	0	0	0	0	0	0	0	0	0	15	15	18	18
RL12-G9	ACAGCACCGTGTTTACTATGAGCA	0	0	0	0	0	0	0	0	0	0	0	0	1	1	0	0	43	43	3327	3316	4427	4417	3851	3848	1	1	0	0	10	10
RL12-G10	CAGACATGACCGCAACAATTATTA	182	182	0	0	2	2	0	0	0	0	0	0	0	0	1340	1338	0	0	0	0	0	0	0	0	123	123	0	0	0	0
RL13-G1	ATCAGCAACCACTTCAACGCTTTC	148	148	0	0	50	50	0	0	0	0	1243	1242	1796	1797	0	0	0	0	861	862	1161	1162	713	714	2763	2763	0	0	1	1
RL13-G2	GCGAATATTAACAACGTTCTCTAC	1736	1731	4336	4336	1318	1319	37	37	0	0	0	0	0	0	0	0	0	0	0	0	0	0	0	0	0	0	0	0	1	1
RL13-G3	AGCATTCATACCACTCCCTGGTTG	0	0	0	0	0	0	0	0	2540	2543	0	0	0	0	0	0	52	52	2	2	2	2	0	0	0	0	0	0	0	0
RL13-G4A	TACAAAGTCGTCGGCAATAACCAC	0	0	0	0	0	0	1884	1891	0	0	0	0	0	0	0	0	2928	2928	0	0	0	0	0	0	0	0	0	0	0	0
RL13-G4B	TACAAAACCGTCGAAAACAACCAC	0	0	0	0	0	0	0	0	0	0	0	0	0	0	0	0	0	0	0	0	0	0	0	0	0	0	10	10	0	0
RL13-G5	AAAAGGCACGCAAATATCCACAAG	0	0	0	0	0	0	0	0	0	0	0	0	0	0	0	0	0	0	0	0	0	0	0	0	0	0	0	0	12	12
RL13-G6	AACATGCGGTCGTAGCTACGAAGT	0	0	60	60	0	0	0	0	0	0	602	602	421	421	0	0	998	998	0	0	0	0	0	0	141	141	0	0	2735	2734
RL13-G7	CAGCTTCATCAACCATACTATCTC	4	4	0	0	0	0	0	0	0	0	0	0	0	0	0	0	0	0	0	0	0	0	0	0	1	1	0	0	11	11
RL13-G8	CAGCGTCGTCAACCATAATATCTC	0	0	0	0	0	0	0	0	1	1	1739	1739	1112	1112	684	684	0	0	0	0	0	0	0	0	0	0	2	2	429	429
RL13-G9	AACCATCCATATATCCACAAAATC	0	0	0	0	0	0	0	0	0	0	0	0	0	0	0	0	0	0	15	15	15	15	21	21	0	0	0	0	0	0
RL13-G10	AACAAAACCGTTGATAGCAACCAC	0	0	0	0	0	0	0	0	0	0	0	0	0	0	2854	2856	0	0	0	0	0	0	0	0	38	38	0	0	0	0
UL1-G1	ATTCCAGTTTCTGGAACTACGGCA	0	0	2	2	0	0	0	0	0	0	1341	1342	1398	1398	0	0	0	0	562	563	1188	1187	721	725	2949	2956	0	0	2	2
UL1-G2	ATTCCAGCTTCTGGAACTATGGCG	3405	3404	4652	4655	826	826	0	0	0	0	0	0	0	0	0	0	0	0	0	0	0	0	0	0	1	1	0	0	0	0
UL1-G3	TCAACATATAGAAACACTCATTAC	0	0	0	0	0	0	0	0	3537	3537	0	0	0	0	0	0	0	0	0	0	0	0	0	0	0	0	0	0	0	0
UL1-G4	CAATAATCCCTGTAATTATTCCAG	2	2	68	68	0	0	1859	1859	1	1	0	0	0	0	0	0	5222	5225	0	0	0	0	0	0	0	0	5990	5989	0	0
UL1-G5	TGACAATCCCTGCAATTATCCAG	0	0	0	0	0	0	0	0	0	0	0	0	0	0	8	13	0	0	0	0	0	0	0	0	0	0	0	0	379	379
UL1-G6	GTAATTCAGGGAGATACAGTTTAT	0	0	45	45	29	29	0	0	0	0	880	880	294	294	0	0	1800	1799	0	0	0	0	0	0	141	141	3	3	2432	2437
UL1-G7	TGCGGTGGACACCGTTTTCTTT	3	3	0	0	0	0	0	0	0	0	0	0	0	0	0	0	0	0	0	0	0	0	0	0	0	0	0	0	56	56
UL1-G8	TGCGGTTGGACACCATTTTACTCG	0	0	0	0	0	0	0	0	0	0	2155	2155	2656	2655	161	161	126	126	0	0	0	0	0	0	0	0	0	0	303	303
UL1-G9	TACAAATACCCATCACAGTAGCTG	0	0	2	2	0	0	0	0	0	0	4	4	1	1	0	0	0	0	3393	3388	4119	4123	3202	3200	0	0	0	0	2	2
UL1-G10	CTAATTCAGGAAGACACAGTTTAT	0	0	0	0	0	0	0	0	0	0	0	0	0	0	5095	5095	0	0	0	0	0	0	0	0	164	164	0	0	0	0
UL9-G1	CCTGTACACAATTCATTAACAATA	3	3	1	1	2	2	2287	2297	0	0	1745	1744	2159	2159	0	0	0	0	1082	1084	1065	1065	630	630	0	0	11	11	3	3
UL9-G2	ACAACATACAGTAGAAACTATTAT	0	0	0	0	0	0	0	0	0	0	0	0	0	0	0	0	0	0	0	0	0	0	0	0	0	0	6604	6601	0	0
UL9-G3	CCCACACAAAAGTCACCCAAAACA	3735	3735	3663	3664	1056	1056	0	0	1845	1847	2330	2330	2465	2465	0	0	2	2	0	0	0	0	1	1	0	0	0	0	1	1
UL9-G4	ACTACATATAGTAGAAATTACTAT	0	0	0	0	6	6	0	0	0	0	65	65	0	0	0	0	0	0	0	0	0	0	0	0	2498	2499	0	0	2099	2099
UL9-G5	CCCGTACGCAGCTCATACACAACA	0	0	0	0	0	0	0	0	0	0	0	0	0	0	0	0	0	0	0	0	0	0	0	0	0	0	0	0	0	0
UL9-G6	TCCACACGTAATTCATTAACGATA	0	0	0	0	0	0	0	0	0	0	880	880	695	695	1	1	1992	1993	0	0	0	0	0	0	0	0	0	0	743	743
UL9-G7	CCCACACATAATCTATTAACAGCA	0	0	45	45	0	0	0	0	1	1	0	0	0	0	0	0	0	0	0	0	0	0	0	0	245	245	0	0	0	0
UL9-G8	CCACACATAATTCATTAACAATGT	0	0	0	0	0	0	0	0	0	0	0	0	0	0	4356	4358	1	1	0	0	0	0	0	0	2	2	0	0	0	0
UL9-G9	TCCACATATAATTCATTAACTATA	1	1	0	0	0	0	1	1	0	0	0	0	0	0	1	1	4025	4014	3818	3824	2887	2892	4072	4072	0	0	0	0	339	339
UL11-G1	CGATGGACTTTAGACACATGTTAC	150	150	285	285	3	3	1763	1763	0	0	3267	3267	2996	2996	1	1	3157	3155	1554	1554	2873	2870	933	931	2821	2821	0	0	7226	7226
UL11-G2	CACATTATCATAAACCTCCCGGAT	0	0	0	0	0	0	0	0	0	0	0	0	0	0	0	0	0	0	0	0	0	0	0	0	0	0	0	0	0	0
UL11-G3	AATCAACATCACCATAAATTTCCC	0	0	9	9	30	30	0	0	0	0	0	0	0	0	0	0	0	0	0	0	0	0	0	0	87	87	0	0	0	0
UL11-G4	CAATTGGCTTCACAGTCGACATCA	0	0	0	0	0	0	0	0	0	0	932	932	1036	1036	1061	1061	1109	1109	0	0	0	0	0	0	0	0	0	0	307	307
UL11-G5	AGTACGCATCGCCATAGAGCTCCC	0	0	0	0	0	0	0	0	0	0	0	0	0	0	0	0	0	0	0	0	0	0	0	0	0	0	3098	3098	0	0
UL11-G6	TCAATGTAACGGAAGAATATACTG	3833	3834	3904	3905	1544	1545	1	1	2316	2313	2580	2585	2602	2602	0	0	0	0	4345	4340	4377	4380	4636	4633	0	0	0	0	9	9
UL11-G7	GACCGCGTTCGTACACCAATCCCA	0	0	0	0	0	0	0	0	1	1	0	0	0	0	5619	5621	0	0	0	0	0	0	0	0	130	130	2	2	0	0
UL73-G1	AACCTCTGCAACTACATCAAAGTC	0	0	0	0	0	0	0	0	9	9	692	692	374	374	0	0	1555	1547	0	0	0	0	0	0	0	0	0	0	297	296
UL73-G2	CCAAGTCCTTCTAGTGTGTCGACG	0	0	0	0	0	0	0	0	0	0	1190	1190	496	487	0	0	0	0	0	0	0	0	0	0	0	0	0	0	0	0
UL73-G3A	AATCGTCCAGTTCTAGCGTGTCAA	0	0	0	0	0	0	0	0	1658	1653	29	29	0	0	490	488	0	0	2863	2858	2055	2053	2745	2748	55	54	2537	2536	7	7
UL73-G3B	CCGAGTCCTCCTAGTTCTAGTGTA	5	5	0	0	0	0	1451	1451	0	0	0	0	0	0	0	0	0	0	0	0	0	0	0	0	0	0	197	197	0	0
UL73-G4A	ACAACTGCAACAACTTCTACAACA	0	0	0	0	0	0	0	0	268	268	0	0	0	0	0	0	3642	3644	0	0	0	0	2	2	0	0	0	0	0	0
UL73-G4B	AGTCCCTCTAGCTCTACTCACACC	1646	1646	2774	2774	1846	1846	0	0	22	22	1397	1398	1316	1316	3733	3733	8	8	0	0	0	0	0	0	2	2	0	0	185	185
UL73-G4C	AGTCCTCTAGCTCTACTCGCACC	0	0	0	0	0	0	0	0	13	13	0	0	0	0	0	0	3	3	0	0	0	0	0	0	0	0	0	0	0	0
UL73-G4D	CGTCCCTCTAGTTCTACTCACGCC	4	4	0	0	0	0	0	0	0	0	0	0	0	0	0	0	0	0	718	718	851	850	478	479	3100	3099	0	0	2080	2079
UL74-G1A	GTACCAAGCCCCTCCAAGGACGAT	0	0	17	17	0	0	0	0	0	0	244	244	229	229	0	0	552	552	0	0	0	0	0	0	0	0	0	0	144	144
UL74-G1B	GTAGACAGTACTTTACCTGACCAG	0	0	3	3	11	11	0	0	2034	2034	0	0	0	0	73	73	75	75	2618	2621	1939	1940	2970	2969	270	270	2297	2297	1	1
UL74-G1C	GTACTGTACCTAACCAGTAACGTC	0	0	0	0	0	0	0	0	0	0	0	0	0	0	0	0	0	0	0	0	0	0	0	0	0	0	0	0	0	0
UL74-G2A	CCATATCCTAGGACGAAATGGTGC	1	2	0	0	0	0	896	896	0	0	0	0	0	0	0	0	0	0	0	0	0	0	0	0	0	0	38	38	0	0
UL74-G2B	GTACTTTACCTATCCAGGGACGTC	0	0	0	0	0	0	0	0	0	0	693	696	934	934	0	0	0	0	0	0	0	0	0	0	11	11	0	0	0	0
UL74-G3	TTTGATAATACCCTGCCATAGGGA	0	0	0	0	2	2	0	0	114	114	2	2	0	0	1	1	2779	2779	0	0	0	0	0	0	0	0	0	0	23	23
UL74-G4	TGACTCTTTACTCGAAAACGCGCT	1256	1259	3665	3667	1369	1369	0	0	0	0	1404	1406	1748	1748	2790	2793	2	2	1	1	0	0	0	0	0	0	0	0	307	307
UL74-G5	TAAGATAGCACGGTATACGGCCAG	0	0	0	0	0	0	0	0	0	0	0	0	0	0	0	0	45	45	380	380	533	534	320	322	2593	2594	0	0	1670	1668
UL120-G1A	TATTGTGTGTTCTTTGATGTACTG	10	10	0	0	0	0	0	0	0	0	56	56	2	2	224	224	0	0	0	0	0	0	0	0	0	0	0	0	0	0
UL120-G1B	ATATTGTGTGTTTTTGGAGTACT	0	0	0	0	0	0	0	0	0	0	0	0	0	0	0	0	0	0	0	0	0	0	0	0	0	0	0	0	0	0
UL120-G2A	TGGTGAGAAAGCGTGTTTTCAACG	2596	2613	2471	2470	1539	1544	0	0	188	188	0	0	0	0	0	0	0	0	0	0	0	0	0	0	0	0	0	0	0	0
UL120-G2B	AAACTGTGTGTTCTGTGGAATACT	0	0	0	0	0	0	0	0	2627	2629	0	0	0	0	0	0	0	0	0	0	0	0	0	0	0	0	0	0	1083	1075
UL120-G3A	CCGTGTATCCCGTGGAATACTGAT	98	97	9	9	23	23	0	0	0	0	0	0	0	0	2319	2317	2328	2333	427	427	586	585	332	332	0	0	3875	3877	0	0
UL120-G3B	TCGTGTGTTATGTGGAATACTGAT	0	0	0	0	0	0	0	0	0	0	0	0	0	0	0	0	2	2	0	0	0	0	0	0	107	107	0	0	0	0
UL120-G4A	GTACGATGCGGAAGGACGATCATC	0	0	0	0	0	0	0	0	0	0	0	0	0	0	0	0	1402	1401	0	0	0	0	0	0	0	0	0	0	0	0
UL120-G4B	TGTGTACGGTGCGGGAGAACGATC	2	2	1	1	0	0	1779	1776	0	0	2682	2677	2511	2508	1	1	71	71	2388	2387	2087	2088	2327	2326	2109	2110	0	0	101	101
UL146-G1	TTCGTTATGTTAACATTATACCAC	0	0	0	0	0	0	2	2	0	0	436	436	270	270	0	0	2502	2502	0	0	2	2	0	0	0	0	3998	3999	137	137
UL146-G2	TTATTCCTTCAGACCTACTAGGGT	0	0	0	0	0	0	1	1	161	161	17	17	0	0	0	0	10	10	0	0	2	2	0	0	0	0	0	0	0	0
UL146-G3	TTATTGTTTGACAGCTATTAAGGT	0	0	0	0	0	0	0	0	0	0	0	0	0	0	0	0	0	0	0	0	0	0	0	0	0	0	0	0	1321	1321
UL146-G4	CCACTAGAAAAGGTAGGCTTAGTA	0	0	0	0	0	0	0	0	0	0	0	0	0	0	0	0	0	0	0	0	0	0	0	0	0	0	0	0	0	0
UL146-G5	CGAGTATTCGATCTTTGTACTACT	0	0	0	0	0	0	0	0	0	0	0	0	0	0	0	0	0	0	0	0	0	0	0	0	0	0	0	0	0	0
UL146-G6	TTAACTTGGTGGAGTAAACGATTT	0	0	0	0	0	0	0	0	0	0	0	0	0	0	0	0	0	0	0	0	0	0	0	0	0	0	0	0	0	0
UL146-G7	TTATTTCCAACGCGGACGTTTGTT	0	0	0	0	0	0	0	0	0	0	0	0	0	0	0	0	0	0	0	0	0	0	0	0	0	0	0	0	0	0
UL146-G8	AGTCGCCAAGGCGGGTGTCTATTG	4019	4017	2905	2901	892	888	1	1	0	0	1	1	0	0	0	0	0	0	2953	2953	2085	2082	2879	2883	1	1	0	0	2	2
UL146-G9	TTAAAATTTTAGTTTCCAAGGCGG	387	387	0	0	3	3	0	0	2175	2175	1509	1509	1029	1029	0	0	1572	1573	623	623	860	860	469	472	0	0	0	0	0	0
UL146-G10	TAATTTCAGTCCTTTTTTCTACTT	0	0	0	0	0	0	0	0	0	0	0	0	0	0	4261	4255	0	0	0	0	0	0	0	0	0	0	0	0	0	0
UL146-G11	ATCCAACCATTTTGATAAATGGTG	6	6	0	0	0	0	0	0	0	0	0	0	0	0	0	0	0	0	0	0	0	0	0	0	3349	3347	0	0	0	0
UL146-G12	CCCAAGCATACGGTCTTGCCTTGG	0	0	0	0	0	0	0	0	0	0	1460	1462	1682	1688	0	0	0	0	0	0	0	0	0	0	150	150	0	0	0	0
UL146-G13	CTAAATCATTATATTTTTTCGACC	0	0	0	0	0	0	0	0	0	0	0	0	0	0	0	0	0	0	0	0	0	0	0	0	0	0	0	0	0	0
UL146-G14	TTATAATTTCCATTTTGGACGTTT	0	0	0	0	0	0	0	0	0	0	0	0	0	0	0	0	0	0	0	0	0	0	0	0	164	164	0	0	0	0
UL139-G1A	GCGCGAGCTGTGGCGCCCCCAAAT	1	1	0	0	1	1	0	0	0	0	0	0	0	0	0	0	0	0	0	0	0	0	0	0	0	0	0	0	0	0
UL139-G1B	GAGCTGTGATACCTCCCAAGTACT	0	0	0	0	0	0	0	0	0	0	0	0	0	0	0	0	0	0	0	0	0	0	0	0	0	0	0	0	0	0
UL139-G2	AAGACGAAGTAGATTGACTGGAAT	0	0	8	8	0	0	0	0	0	0	806	806	470	470	0	0	0	0	3594	3593	2660	2660	4011	4013	0	0	0	0	6	6
UL139-G3	AATTATTGCCGCCAAAACCATTAA	0	0	0	0	0	0	0	0	0	0	0	0	0	0	4049	4049	3559	3551	2	2	0	0	0	0	87	87	0	0	40	40
UL139-G4	ACTGCCGTTAGAGGCATTAATACA	0	0	0	0	0	0	1172	1179	0	0	0	0	0	0	0	0	1298	1298	0	0	0	0	0	0	0	0	0	0	634	634
UL139-G5	ACCAGGTACCGTTTGTGCCATCGA	6	6	1	1	0	0	0	0	2334	2331	0	0	0	0	62	62	0	0	0	0	0	0	0	0	3527	3530	3670	3671	182	187
UL139-G6	AATTACTGCCGCCAAAAGCTTGAA	0	0	0	0	0	0	0	0	0	0	0	0	0	0	0	0	0	0	0	0	0	0	0	0	0	0	0	0	0	0
UL139-G7	AATTACTGCCACCAAAGGCTTGAA	1464	1464	2428	2426	969	970	98	98	0	0	0	0	0	0	0	0	0	0	0	0	0	0	0	0	216	216	1	1	0	0
UL139-G8	GAGTTGTGGGACTGTCCAATTACT	0	0	0	0	0	0	1	1	0	0	1092	1092	1028	1028	46	46	0	0	0	0	0	0	0	0	0	0	0	0	1	1
RL5A-G1-D1	TGTACCATTATTAGTCACCACCGT	4145	4145	3444	3442	1659	1657	0	0	0	0	0	0	0	0	0	0	0	0	0	0	0	0	0	0	0	0	0	0	0	0
RL5A-G1-D2-1	GAAACACAGACCGAGCGTTGTACC	0	0	65	65	48	48	0	0	0	0	3	3	8	8	0	0	5	5	0	0	0	0	0	0	180	180	0	0	0	0
RL5A-G1-D2-2	GAAACACAGACCGCGCGTTGTACC	358	358	4	4	72	72	0	0	0	0	1924	1921	2689	2683	0	0	5853	5858	0	0	1	1	0	0	0	0	1	1	0	0
UL111A-D1	ATCGAGTAAAACGGTCTTTCTTTT	0	0	0	0	0	0	0	0	0	0	0	0	0	0	0	0	0	0	0	0	0	0	0	0	0	0	0	0	0	0
UL111A-WT	ATCGAGTAAAACCTACGTTGGTAG	1060	1041	2551	2555	1247	1243	1198	1198	2242	2242	2120	2121	2499	2501	1751	1752	2518	2517	2183	2180	1913	1913	2230	2231	2736	2736	2137	2135	1761	1761
US9-D1-1	TTAGCCTCTTCCTCGAACCCAGGG	0	0	0	0	26	26	1663	1663	1433	1433	2459	2459	2262	2266	1405	1404	678	677	473	474	610	610	438	438	184	184	2826	2826	261	261
US9-D1-2	TTAGCCTCTTCCTTGAACCCAGGG	0	0	2	2	0	0	0	0	0	0	6	6	2	2	1	1	17	17	2064	2062	2044	2038	2226	2222	0	0	1	1	1308	1309
US9-WT	TCAATCGTCTTTAGCCTCTTCTTC	1613	1613	1949	1951	1306	1305	0	0	1	1	2	1	1	1	229	230	2147	2145	3	2	4	4	1	1	2651	2651	91	91	0	0

[a]Values for datasets that meet threshold criteria (1) to (4) or that meet criteria (1) to (3) and are 10-20 for criterion (4) are in black font; values for other datasets are in red font.
[b]Values in rows 20-23 that meet quality threshold criteria (1) to (4) are shaded dark green.
[c]Values in rows 25-56 and 59-166 that meet threshold criteria are shaded dark green or dark gold; WT (rows 167-174), wild type; D (rows 167-174), deletion.
