# Supplementary material for: Multiple-Strain Infections of Human Cytomegalovirus With High Genomic Diversity Are Common in Breast Milk From Human Immunodeficiency Virus–Infected Women in Zambia
Source: J Infect Dis. 2019 May 3;220(5):792–801. doi: 10.1093/infdis/jiz209 (PMC6667993; doi:10.1093/infdis/jiz209)
Supplement: jiz209_suppl_Supplementary_Table_2 [file jiz209_suppl_supplementary_table_2.pdf]

Supplementary Table 2. Congruence of results obtained using short and long motifs.

SUMMARY DATA[a]

GENOTYPING DATA[b]

Donor	193						141						173						174					
Dataset	193R16						141R16						173R16						174L16					
Motif	UL73-5'	UL73	UL74-5'	UL74-C	UL74-3'	UL74	UL73-5'	UL73	UL74-5'	UL74-C	UL74-3'	UL74	UL73-5'	UL73	UL74-5'	UL74-C	UL74-3'	UL74	UL73-5'	UL73	UL74-5'	UL74-C	UL74-3'	UL74
G1-G1A	0	0	64	2	1	0	0	0	5	3	0	0	317	320	648	902	577	487	63	90	14	11	3	49
G2-G2B	0	0	0	1	15	0	345	330	605	1675	458	477	77	26	57	110	246	51	583	506	718	907	557	732
G3A-G1B	5224	4853	5457	4628	5147	5321	1009	268	502	1979	286	492	1138	1105	2049	2216	2104	1530	60	57	1	34	2	0
G3B-G2A	0	0	3	1	55	0	0	0	1	4	11	0	0	0	46	47	122	42	0	0	4	4	9	2
G4A-G3	0	0	0	14	4	0	0	0	1	6	3	0	1734	1636	785	896	1492	580	989	934	1759	1165	963	1597
G4B-G4	47	39	7	7	3	0	0	0	7	5	0	0	212	168	4	35	542	559	109	54	3	7	47	137
G4C-G1C	1	0	1	1	35	0	1	0	2	0	3	0	5	1	3	2	10	1	4	0	1	123	7	0
G4D-G5	16	0	18	9	19	0	13	15	2	3	4	0	6	0	11	8	15	0	1	0	2	9	1	0

Donor	174						243																	
Dataset	174R16						243L16						243R4						243R16					
Motif	UL73-5'	UL73	UL74-5'	UL74-C	UL74-3'	UL74	UL73-5'	UL73	UL74-5'	UL74-C	UL74-3'	UL74	UL73-5'	UL73	UL74-5'	UL74-C	UL74-3'	UL74	UL73-5'	UL73	UL74-5'	UL74-C	UL74-3'	UL74
G1-G1A	26	26	21	102	23	10	0	0	15	2	0	0	0	0	45	5	3	17	0	0	25	0	3	0
G2-G2B	216	163	522	630	384	233	0	0	0	3	3	0	3	0	8	3	9	0	0	0	0	0	4	0
G3A-G1B	26	24	49	109	2	110	140	0	4	1	5	0	2	0	23	11	15	3	0	0	0	5	15	11
G3B-G2A	0	0	1	3	6	0	7	5	7	7	9	2	0	0	7	19	53	0	0	0	0	0	9	0
G4A-G3	1128	1013	1018	850	1022	765	1	0	4	5	2	0	1	0	2	30	7	0	0	0	2	5	1	2
G4B-G4	3	6	1	19	30	0	2049	1646	31	2826	3936	1259	3121	2774	8	3309	3173	3667	2077	1846	12	1427	972	1369
G4C-G1C	0	0	1	3	4	0	1	0	2	1	2	0	2	0	1	5	9	0	0	0	2	1	4	0
G4D-G5	0	0	3	3	14	0	8	4	10	4	16	0	13	0	31	8	45	0	2	0	3	11	5	0

Donor	248						258						259											
Dataset	248R4						258R4						259L16						259R16					
Motif	UL73-5'	UL73	UL74-5'	UL74-C	UL74-3'	UL74	UL73-5'	UL73	UL74-5'	UL74-C	UL74-3'	UL74	UL73-5'	UL73	UL74-5'	UL74-C	UL74-3'	UL74	UL73-5'	UL73	UL74-5'	UL74-C	UL74-3'	UL74
G1-G1A	0	0	4	3	0	0	100	9	30	1	3	0	754	692	343	396	434	244	399	374	364	381	251	229
G2-G2B	0	0	2	0	7	0	2	0	1	4	4	0	1364	1190	1065	1083	1615	696	552	487	871	1091	1462	934
G3A-G1B	1	0	0	2	1	0	1798	1653	2233	2359	2356	2034	41	29	11	13	12	0	2	0	20	44	17	0
G3B-G2A	1553	1451	614	1161	1206	896	0	0	2	4	29	0	1	0	6	11	78	0	0	0	6	13	73	0
G4A-G3	1	0	3	0	2	0	331	268	95	302	397	114	2	0	6	5	65	2	1	0	2	9	13	0
G4B-G4	0	0	16	3	1	0	21	22	4	3	0	0	1526	1398	15	1478	1481	1406	1527	1316	23	1735	1655	1748
G4C-G1C	1	0	0	3	6	0	17	13	1	1	1	0	5	0	12	4	10	0	3	0	6	7	7	0
G4D-G5	2	0	4	8	5	0	5	0	4	11	7	0	20	0	24	22	52	0	17	0	33	15	42	0

Donor	264						277						278											
Dataset	264L16						277R16						278L16						278R4					
Motif	UL73-5'	UL73	UL74-5'	UL74-C	UL74-3'	UL74	UL73-5'	UL73	UL74-5'	UL74-C	UL74-3'	UL74	UL73-5'	UL73	UL74-5'	UL74-C	UL74-3'	UL74	UL73-5'	UL73	UL74-5'	UL74-C	UL74-3'	UL74
G1-G1A	0	0	19	1	5	0	1738	1547	860	1350	1658	552	1	0	22	3	5	0	0	0	27	4	2	0
G2-G2B	0	0	4	10	9	0	1	0	1	8	68	0	1	0	8	2	16	0	4	0	8	3	10	0
G3A-G1B	531	488	315	809	667	73	0	0	120	78	20	75	3229	2858	3125	2560	3168	2621	2382	2053	2170	1929	1998	1940
G3B-G2A	0	0	5	7	23	0	0	0	1	5	33	0	0	0	4	17	60	0	1	0	7	10	43	0
G4A-G3	0	0	2	2	4	1	3788	3644	2850	3408	3381	2779	1	0	7	26	7	0	2	0	2	21	11	0
G4B-G4	4557	3733	28	2881	3107	2793	1	8	8	6	0	2	4	0	11	7	0	1	2	0	8	7	3	0
G4C-G1C	2	0	1	9	9	0	5	3	5	4	3	0	3	0	7	4	39	0	2	0	3	2	45	0
G4D-G5	7	0	5	3	10	0	13	0	58	15	11	45	819	718	454	547	644	380	931	850	607	449	534	534

Donor	278						281						283						288					
Dataset	278R16						281R4						283R16						288R4					
Motif	UL73-5'	UL73	UL74-5'	UL74-C	UL74-3'	UL74	UL73-5'	UL73	UL74-5'	UL74-C	UL74-3'	UL74	UL73-5'	UL73	UL74-5'	UL74-C	UL74-3'	UL74	UL73-5'	UL73	UL74-5'	UL74-C	UL74-3'	UL74
G1-G1A	0	0	16	9	4	0	0	0	15	5	1	0	1	0	12	2	1	0	314	296	78	53	4	144
G2-G2B	2	0	5	7	13	0	0	0	16	122	16	11	0	0	2	2	10	0	0	0	5	3	11	0
G3A-G1B	3090	2748	3308	2907	3109	2969	176	54	248	390	209	270	2903	2536	2741	3008	5072	2297	10	7	13	3	13	1
G3B-G2A	0	0	2	10	67	0	4	0	8	10	43	0	196	197	3	6	48	38	0	0	3	8	31	0
G4A-G3	2	2	6	38	8	0	0	0	0	18	5	0	0	0	4	6	11	0	107	0	43	191	7	23
G4B-G4	4	0	10	7	3	0	2	2	9	11	1	0	0	0	7	6	0	0	185	185	6	13	330	307
G4C-G1C	4	0	6	4	34	0	2	0	3	6	19	0	1	0	1	1	1	0	2	0	4	4	4	0
G4D-G5	572	479	477	460	392	322	3423	3099	2080	1984	4086	2594	15	0	5	17	23	0	2226	2079	1765	1858	2178	1668

[a]For datasets that meet the quality thresholds (Supplementary Table 1).

[b]Short and long motif headings are shown in light and dark gold font, respectively (Table 1). In rows 7-14, UL73-UL74 linkages are listed (Figure 1) and values that meet the threshold criteria are shaded dark gold.
