# Supplementary material for: Multiple-Strain Infections of Human Cytomegalovirus With High Genomic Diversity Are Common in Breast Milk From Human Immunodeficiency Virus–Infected Women in Zambia
Source: J Infect Dis. 2019 May 3;220(5):792–801. doi: 10.1093/infdis/jiz209 (PMC6667993; doi:10.1093/infdis/jiz209)
Supplement: jiz209_suppl_Supplementary_Table_3 [file jiz209_suppl_supplementary_table_3.pdf]

Supplement Table 3.

HCMV genotypes assigning haplotypes: diverse between individuals and prevalence shows restricted genetic linkage

				Genotypes hypervariable genes[a]											
ID[b]	Mix[b]	Strain[b]	HIV	RL5A	RL6	**RL12[c]**	**RL13[c]**	**UL1[c]**	UL9	UL11	**UL73[c]**	**UL74[c]**	UL120	UL146	UL139
**154**	major	z-a	-	4	7	4A	4A	4	1	1	4A	3	4B	13	5
166	single	z-b	-	-	3	9	-	-	9	-	-	1A	4A	-	3
**193**	single	z-c	-	1	3	8	8	8	4	1	3A	1B	2B	12	3
232	major	z-d	-	1	1	1A	8	-	6	4	4D	5	4B	14	7
239	single	z-e	-	6	1	10	-	-	9	-	4B	-	2A	2	7
263	single	z-f	-	-	2	10	10	10	8	7	3A	1B	2A	-	4
280	single	z-g	-	1	-	-	6	6	7	-	1	1A	-	-	-
**141**	major	z-h	+	1	3	4B	2	4	2	6	2	2B	4B	12	3
**141**	minor	z-i	+	6	2	2	2	2	3	5	3A	1B	3B	2	8
173	major	z-j	+	1	*3	*8	*8	8	*4	1	*3A	*1B	1A	9	1A
173	minor	z-k	+	2	*2	*6	*6	*6	*8	*3	*4A	*3	4B	*3	*7
**174**	major	z-l	+	2	4	1B	1	1	4	1	4A	3	2B	9	5
**174**	minor	z-m	+	1	2	6	6	6	6	4	2	2B	3A	7	7
181	major	z-n	+	1	2	4A	*6	6	6	1	4A	3	*2A	8	4
**243**	major	z-o	+	1	6	2	2	2	3	6	4B	4	2A	8	7
**248**	major	z-p	+	1	1	4A	4A	4	1	1	3B	2A	4B	-	4
**258**	major	z-q	+	6	2	3	3	3	3	6	3A	1B	2B	9	2
**259**	major	z-r	+	1	3	8	8	8	3	1	4B	4	4B	9	8
**259**	minor	z-s	+	2	4	1A	1	1	1	6	2	2B	1A	12	2
**264**	major	z-t	+	1	2	10	10	10	8	7	4B	4	3A	10	3
**264**	minor	z-u	+	1	1	8	8	8	8	4	3A	1B	1A	10	5
**277**	major	z-v	+	1	3	4A	4A	4	9	1	4A	3	3A	1	3
**277**	minor	z-w	+	1	3	6	6	6	6	4	1	1A	4A	9	4
**278**	major	z-x	+	6	3	9	9	9	9	6	3A	1B	4B	8	2
**278**	minor	z-y	+	2	4	1A	1	1	1	1	4D	5	3A	9	2
**281**	major	z-z	+	6	3	1A	1	1	4	1	4D	5	4B	1	5
281	minor	z-a1	+	1	3	*6	*6	*6	7	*3	3A	1B	3B	*1	5
**283**	major	z-b1	+	-	4	4B	4B	4	2	5	3A	1B	3A	1	5
**288**	major	z-c1	+	2	4	6	6	6	4	1	4D	5	2B	3	4
288	minor	z-d1	+	1	3	7	8	*8	*6	4	1	1A	4B	1	5
Number genotypes /total				4/6	6/7	10/12	10/11	8/11	8/9	6/7	7/8	7/8	7/8	10/14	7/9
Prevalence key			>35%		>30%	>25%		>20%		>15%		>10%		>5%	>1%

[a]Genotypes determined by long motifs for 12 genes. The genotype prevalence is indicated by the prevalence key heat map was calculated using Excell for each hypervariable gene by measuring the relative frequency of each genotype observed in the cohort population shown.

[b]Cut-offs used were as described in [22], minimum 10 reads per SNP, depth of at least 100 and frequency >2%. Major or minor strains were identified by >60% or <40% genotype prevalence, respectively. *Amibguous due to genotypes with similar prevalence, - reads too low, thus unable to haplotype; **Bold ID** are only assigned haplotypes.

[c]Linked hypervariable genes were indicated by those genotypes with the same relative frequency as indicated in bold by UL73/UL74 and RL12/RL13/UL1.
